# Antioxidant Nutrients and Risk of Latent Autoimmune Diabetes in Adults and Type 2 Diabetes: A Swedish Case-Control Study and Mendelian Randomization Analysis

**DOI:** 10.3390/nu15112546

**Published:** 2023-05-30

**Authors:** Anna-Maria Lampousi, Josefin E. Löfvenborg, Emma Ahlqvist, Tiinamaija Tuomi, Alicja Wolk, Sofia Carlsson

**Affiliations:** 1 Institute of Environmental Medicine, Karolinska Institutet, 171 77 Stockholm, Sweden; 2 Department of Risk and Benefit Assessment, Swedish Food Agency, 751 26 Uppsala, Sweden; 3 Department of Clinical Sciences, Lund University, 214 28 Malmö, Sweden; 4 Institute for Molecular Medicine Finland (FIMM) and Research Programs Unit, Clinical and Molecular Metabolism, University of Helsinki, 00014 Helsinki, Finland; 5 Department of Endocrinology, Helsinki University Hospital, 00029 Helsinki, Finland; 6 Folkhälsan Research Center, 00250 Helsinki, Finland

**Keywords:** antioxidants, autoimmune diabetes, LADA, type 2 diabetes

## Abstract

Antioxidant vitamins C and E are inversely associated with type 1 diabetes (T1D). We investigated if antioxidants are also associated with latent autoimmune diabetes in adults (LADA), with low (LADA^low^) and high (LADA^high^) autoantibody levels, type 2 diabetes (T2D), and estimates of beta cell function (HOMA-B) and insulin resistance (HOMA-IR). We used Swedish case-control data with incident cases of LADA (*n* = 584) and T2D (*n* = 1989) and matched population-based controls (*n* = 2276). Odds ratios (OR) and 95% confidence intervals (CI) were calculated per one standard deviation higher beta-carotene, vitamin C, vitamin E, selenium, and zinc intakes. Two-sample Mendelian randomization (MR) analyses assessed causality between genetically predicted circulating antioxidants and LADA, T1D, and T2D, using summary statistics from genome-wide association studies. Among the antioxidants, vitamins C and E were inversely associated with LADA^high^ (OR 0.84, CI 0.73, 0.98 and OR 0.80, CI 0.69, 0.94 respectively), but not with LADA^low^ or T2D. Vitamin E was also associated with higher HOMA-B and lower HOMA-IR. MR analyses estimated an OR of 0.50 (CI 0.20, 1.25) for the effect of vitamin E on T1D, but did not support causal relationships between antioxidants and either LADA or T2D. In conclusion, vitamin E may have a protective effect on autoimmune diabetes, possibly through preserved beta cell function and less insulin resistance.

## 1. Introduction

Latent autoimmune diabetes in adults (LADA) may be the most common form of autoimmune diabetes in adults and is estimated to account for 3–12% of all diabetes [1]. It is referred to as a hybrid form of diabetes, since it combines the characteristics of type 1 and type 2 diabetes, including both islet autoimmunity and insulin resistance [2]. Regarding genetic susceptibility, LADA resembles type 1 diabetes with most of the genetic risk being conferred by HLA haplotypes [3]. Knowledge about modifiable risk factors is limited, but the findings of the small number of studies conducted to date suggest that dietary factors may be of importance in the development of LADA [4].

There is some indication that a high intake or blood level of antioxidants, specifically vitamin C and vitamin E, may reduce the risk of type 1 diabetes [5]. A plausible mechanism involves a decrease in oxidative stress, a process that can damage the insulin-producing beta cells and lead to impaired beta cell function and insulin resistance [6]. Dietary antioxidants act synergistically with our body’s endogenous antioxidants (e.g., glutathione, superoxide dismutase, and others) to ensure redox homeostasis [7]. In particular, having a strong antioxidant defence can counteract the excess production of highly reactive molecules, known as free radicals, which are generated due to various environmental exposures, including smoking, and cause oxidative stress [8]. Furthermore, total antioxidant intake is inversely associated with type 2 diabetes and insulin resistance [9], but findings on individual antioxidants are inconsistent [10,11]. Considering this evidence, dietary antioxidants may also play a protective role in the incidence of LADA, but this has not been investigated. LADA is a heterogeneous disease, i.e., those with higher autoimmunity (LADA^high^) are more likely to encounter an impaired beta cell function, similar to type 1 diabetes, while those with less autoimmunity (LADA^low^) may have a phenotype similar to type 2 diabetes [12,13,14]. This heterogeneity is important to consider since the role of diet may vary across subtypes.

We investigated whether the intake of antioxidant nutrients, namely beta-carotene, vitamin C, vitamin E, selenium, and zinc, reduces the risk of LADA with varying degrees of autoimmunity, or type 2 diabetes. We additionally examined if such intakes were associated with beta cell function or insulin resistance. To this aim, we used data from a Swedish population-based study with incident cases. We also used a two-sample Mendelian randomization design to assess whether circulating beta-carotene, vitamin C, and vitamin E are causally linked to LADA, type 1, or type 2 diabetes.

## 2. Materials and Methods

### 2.1. The ESTRID Study

#### 2.1.1. Study Population and Design

We analyzed data from the Swedish population-based case-control Epidemiological Study of Risk Factors for LADA and Type 2 Diabetes (ESTRID) that is nested within the All New Diabetics in Scania (ANDIS) register and biobank [15]. Incident diabetes cases were recruited through ANDIS and the All New Diabetics in Uppsala (ANDiU) register, which aim at characterizing all new diabetes cases in terms of clinical and genetic features in the counties of Scania and Uppsala, respectively. Since 2010, we invite all newly diagnosed LADA cases and a random sample of incident type 2 diabetes cases (four per LADA case) to participate in ESTRID, together with a random sample of diabetes-free individuals ≥35 years (six per LADA case) who were identified through the Swedish population registry and matched to the cases by time and region (incidence density sampling). Type 1 diabetes cases are not invited to ESTRID and, thus, we did not include observational data on this outcome. Eligible for the present study were cases and controls included in ESTRID between 2010 and 2019 that reported plausible energy intakes (within three standard deviations from the log_e_-transformed sex-specific mean energy intake in controls). After excluding 199 individuals with implausible energy intake, the current analytical sample consists of 584 LADA cases, 1989 type 2 diabetes cases, and 2276 controls (Figure S1).

#### 2.1.2. Laboratory Analyses and Diabetes Classification

Diabetes cases were diagnosed within the healthcare sector and provided blood samples for further classification and analysis [15]. Specifically, autoantibodies against glutamic acid decarboxylase (GADA) were analyzed with the enzyme-linked immunosorbent assay (RSR, Cardiff, UK), with 84% sensitivity and 98% specificity at 10.7 U/mL cut-off [16] and maximum value at 250 U/mL. In addition, serum C-peptide was analyzed by the IMMULITE 2000 immunoassay system (Siemens Healthcare Diagnostics Product Ltd., Llanberis, UK) or by Cobas e601 analyzer (Roche Diagnostics, Mannheim, Germany). In line with the criteria proposed by the Immunology of Diabetes Society [17], LADA classification included age ≥35 years, GADA positivity (≥10 U/mL), and C-peptide ≥ 0.2 nmol/L (IMMULITE) or ≥0.3 nmol/L (Cobas) to be distinguished from type 1 diabetes cases. For analytic purposes, LADA cases were subclassified based on median GADA levels as LADA^low^ (<250 U/mL) and LADA^high^ (≥250 U/mL). The type 2 diabetes definition included age ≥35 years, GADA negativity (<10 U/mL), and C-peptide > 0.6 nmol/L (IMMULITE) or >0.72 nmol/L (Cobas). The homeostasis model assessment (HOMA) was used for estimating beta cell function (HOMA-B) and insulin resistance (HOMA-IR) based on fasting plasma glucose and serum C-peptide levels at the time of diagnosis [18].

#### 2.1.3. Genetic Risk Assessment

Blood samples were analyzed for genotyping of cases using iPlex (Sequenom, San Diego, CA, USA) or TaqMan assays (Thermo Fisher Scientific, Carlsbad, CA, USA) at the Clinical Research Center in Malmö, Sweden [15]. Missing genetic variants were imputed using Infinium CoreExome v1.1 (Illumina, San Diego, CA, USA), based on the Haplotype Reference Consortium (version r1.1 2016). Three single nucleotide polymorphisms (SNPs) (rs3104413, rs2854275, rs9273363) were used for predicting HLA-DR and HLA-DQ genotypes relevant to autoimmune diabetes, with 99.3% accuracy [19]. High-risk HLA genotypes were DR4-DQ8, DR3-DQ2, DR3/4, DR3/3, and DR4/4, while low/intermediate risk genotypes were DR4-DQ7, DR3/X, DR4/X, and DRX/X (where X ≠ DR3 or DR4) [19].

#### 2.1.4. Dietary and Covariate Assessment

Habitual diet over the past year, which was the year before diagnosis among cases, was assessed with a 132-item semi-quantitative food frequency questionnaire (FFQ). Frequencies were prespecified and ranged between “0 times/month” and “≥3 times/day”, except for some drinks (i.e., milk, sweetened beverages, coffee, tea), sugar, cheese, and bread, for which participants could indicate the exact frequencies. Energy (kcal/day) and nutrient intakes were estimated by multiplying the consumption frequency of each item by its nutrient content, according to the Swedish National Food Agency database, applying age- and sex-specific portion sizes. Nutrient intakes were adjusted for total energy using the residual method [20]. The FFQ has been validated for nutrient estimation using repeated 24-h recalls [21], with Spearman correlation coefficients of 0.51 for beta-carotene (mg/day), 0.81 for vitamin C (mg/day), 0.57 for vitamin E (alpha-tocopherol) (mg/day), 0.75 for selenium (µg/day), and 0.56 for zinc (mg/day). Participants were additionally asked if they had ever consumed dietary supplements for at least three months, including beta-carotene, vitamin C, vitamin E, selenium, zinc, and multivitamins. From the FFQ, we also derived information on potentially important dietary covariates, i.e., red meat, fish, sweetened beverage, and coffee intakes (g/day), which were categorized based on the tertiles among controls, and alcohol consumption, which was categorized into none, 0.001 to <4.9 g/day, 4.9 to <14.9 g/day, 14.9 to <24.9 g/day, and ≥24.9 g/day.

Further information on lifestyle, as well as demographic and clinical characteristics, was obtained through a comprehensive questionnaire. Leisure-time physical activity during the past year (controls) or the year before diagnosis (cases) was categorized into four levels, i.e., inactive, mildly active, active, and very active. Smoking status was classified into never, ex, or current smoking. Highest educational attainment was categorized into primary, intermediate, and university. Weight and height were used to calculate body mass index (BMI). Cardiovascular disease diagnosis, hypertension, and family history of diabetes were also reported.

### 2.2. Mendelian Randomization

We performed two-sample Mendelian randomization (MR) analyses using publicly available summary statistics from a European genome-wide association study (GWAS) of LADA (2634 cases and 5947 controls) [22], and GWAS meta-analyses of type 1 diabetes including 12 European studies (9266 cases and 15,574 controls) [23] and of type 2 diabetes including 32 European studies (80,154 cases and 853,816 controls) [24] (Figure S2). As instrumental variables for antioxidant nutrients, we used SNPs independently (not in linkage disequilibrium, R^2^  < 0.01) associated with circulating beta-carotene (2 SNPs), vitamin C (ascorbate [10 SNPs] or ascorbate metabolites [14 SNPs]), and vitamin E (alpha-tocopherol metabolites [11 SNPs] or gamma-tocopherol metabolites [13 SNPs]) in the largest or uniquely available GWAS in populations of European ancestry to date (Table S1). Further information about the genetic instruments for circulating antioxidants can be found in Appendix A.1. We did not perform MR analyses for circulating selenium and zinc, since these exposures were already addressed in MR studies of type 2 diabetes and were not associated with either LADA or type 1 diabetes in observational analyses.

### 2.3. Statistical Analysis

All statistical analyses were performed with Stata Statistical Software Release 16 (StataCorp, College Station, TX, USA). Participant characteristics were described as means with standard deviation (SD) or medians with interquartile range (IQR) for continuous variables, or as proportions for categorical variables, and were compared between LADA and type 2 diabetes cases based on the *p*-values obtained by Student *t*-test, Kruskal–Wallis H test, and χ^2^ test, respectively.

We estimated odds ratios (OR) and 95% confidence intervals (CI) of LADA, LADA^high^, LADA^low^, and type 2 diabetes in relation to quartiles of and per 1 SD increase in antioxidant nutrient intakes and for any vs. no use of antioxidant nutrient supplements, using conditional logistic regression models. Model 1 was adjusted for age and sex; Model 2 was further adjusted for education, physical activity, smoking, alcohol intake, family history of diabetes, and BMI; Model 3 was further adjusted for fatty fish (except for selenium), red meat (except for selenium and zinc), sweetened beverages, and coffee intake; and Model 4 was further adjusted for history of cardiovascular diseases or hypertension. In the results section we will present estimates based on Model 4 unless otherwise stated. Separate analyses were performed for LADA cases with low/intermediate- and high-risk HLA genotypes. Adjusted (Model 4) linear regression models were used to estimate the association between 1 SD higher antioxidant nutrient intakes and log_e_-transformed levels of HOMA-B and HOMA-IR.

To investigate if there is a causal relationship between circulating antioxidants and LADA, type 1, or type 2 diabetes in MR analyses, we used the inverse-variance weighted method, followed by sensitivity analyses with other MR estimators, i.e., MR-Egger, weighted median, and weighted mode (Appendix A.2. Statistical Analysis). The *p*-value of Cochran’s Q statistic was used to examine heterogeneity between genetic instruments, where Q > K – 1 (K = number of instruments) indicates the presence of heterogeneity and, accordingly, evidence of pleiotropy. The I^2^ statistic was used to quantify heterogeneity, with I^2^ ≥ 50% being indicative of substantial heterogeneity.

### 2.4. Sensitivity Analyses

To assess whether risk estimates are independent of other antioxidant nutrients, we performed sensitivity analyses by further adjusting the regression models for the use of antioxidant supplements and mutually adjusting all the studied antioxidants. Effect modification by smoking status was assessed by stratifying analyses for current smokers and non-smokers. To reduce the risk of exposure misclassification, we performed sensitivity analyses excluding individuals who had consumed dietary supplements of antioxidant nutrients (*n* = 1240, 26%) and cases that had radically changed their diet after diagnosis (*n* = 758, 29%) or had diabetes for a duration of longer than three months (*n* = 1826, 71%) when responding to the FFQ.

## 3. Results

### 3.1. Participant Characteristics

The mean age at inclusion was 59.0 (SD 13.8) years in controls, 59.1 (SD 12.4) years in LADA, and 63.3 (SD 10.4) years in people with type 2 diabetes. Compared to individuals with type 2 diabetes, those with LADA, and particularly LADA^high^, had lower HOMA-IR and HOMA-B and were more likely to be treated with insulin and have a high-risk HLA genotype (Table 1). History of cardiovascular diseases or hypertension, as well as family history of diabetes, were more prevalent among people with type 2 diabetes than LADA. In addition, LADA^high^ individuals had a lower BMI and were more likely to smoke, consume alcohol, and have a higher education than those LADA^low^.

Participant characteristics and intakes of different food items across quartiles of antioxidant nutrient intakes are presented in Tables S2–S6. In brief, individuals with the highest consumption of beta-carotene and vitamin C had a healthier lifestyle (e.g., physically active, non-smokers) and were more likely to consume fruits, vegetables, and whole grains, and less likely to consume meat, dairy, and sweetened beverages. High intake of vitamin E was characterized by a higher intake of nuts, whole grains, and alcohol, and a lower intake of meat, dairy, and sweetened beverages, whereas no clear difference was seen for other lifestyle factors. Those with the highest consumption of selenium and zinc were less likely to be women and to consume fruits, nuts, and sweetened beverages, and had higher intakes of whole grain, meat, and alcohol. In addition, those with the highest selenium intakes also had the highest fish consumption.

### 3.2. Antioxidant Nutrient Intakes and Risk of LADA

**Figure 1:**
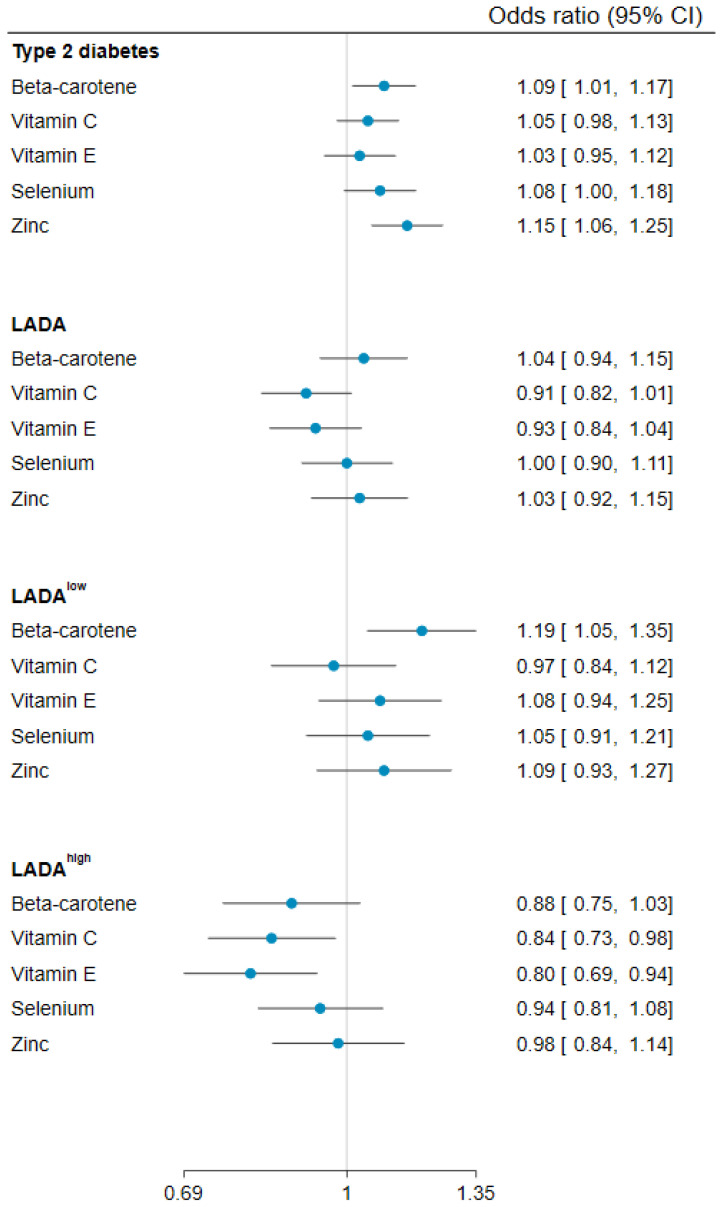
ORs and 95% Cis of type 2 diabetes, LADA, LADA^low^, and LADA^high^ in relation to 1 SD higher antioxidant nutrient intakes. ORs adjusted for age, sex, education, physical activity, smoking, intake of alcohol, family history of diabetes, BMI, tertiles of fatty fish (except for selenium), red meat (except for selenium and zinc), sweetened beverages, and coffee intake, and history of cardiovascular diseases or hypertension.

Overall, the associations between LADA and the antioxidants were weak (Figure 1, Table 2), except for a reduced risk observed in relation to vitamin C (OR for high vs. low intake: 0.66, 95% CI 0.49, 0.88). These results were consistent across models adjusted for different sets of covariates (Table S7). Stratifying LADA by GADA levels revealed inverse associations between vitamins C and E and LADA^high^, but not LADA^low^ (Figure 1, Table 2); ORs for LADA^high^ were estimated at 0.51 (95% CI 0.35, 0.76) for high vs. low intake and at 0.84 (95% CI 0.73, 0.98) per 1 SD increase in vitamin C. Corresponding estimates for vitamin E were 0.66 (95% CI 0.45, 0.97) and 0.80 (95% CI 0.69, 0.94), respectively. There was also a positive association between intake of beta-carotene and LADA^low^ (OR for high vs. low intake: 1.62, 95% CI 1.10, 2.40) (Figure 1, Table 2). Regarding dietary supplements, no inverse associations were observed with the supplementation of individual antioxidant nutrients. However, intake of multivitamins was associated with a lower risk of LADA (OR 0.71, 95% CI 0.54, 0.92).

### 3.3. Antioxidant Nutrient Intakes and Risk of Type 2 Diabetes

Vitamin C and vitamin E intakes were not associated with type 2 diabetes. Conversely, a higher risk of type 2 diabetes was noted in relation to higher beta-carotene, selenium, and zinc intakes (Figure 1, Table 2), with OR 1.09 (95% CI 1.01, 1.17), 1.08 (95% CI 1.00, 1.18), and 1.15 (95% CI 1.06, 1.25) per 1 SD higher intake, respectively. Intake of multivitamins was inversely associated with type 2 diabetes (OR: 0.79, 95% CI 0.61, 0.93), but no associations were observed with supplementation of individual antioxidant nutrients.

### 3.4. Antioxidant Nutrient Intakes and HOMA-B or HOMA-IR

There was a positive association between the intake of vitamin E and HOMA-B in both LADA (β 0.100, 95% CI 0.021, 0.179) and type 2 diabetes (β 0.027, 95% CI 0.000, 0.054) (Table 3). This corresponds to a 10% increase in HOMA-B in LADA and 3% in type 2 diabetes per 1 SD higher vitamin E intake. Intake of vitamin E was also inversely associated with HOMA-IR in type 2 diabetes (β −0.037, 95% CI −0.065, −0.009), while the association was similar but not significant in LADA (β −0.077, 95% CI −0.156, 0.003). None of the remaining antioxidants were associated with either HOMA-B or HOMA-IR.

### 3.5. Mendelian Randomization Analyses of Antioxidants and LADA, Type 1, and Type 2 Diabetes

**Figure 2:**
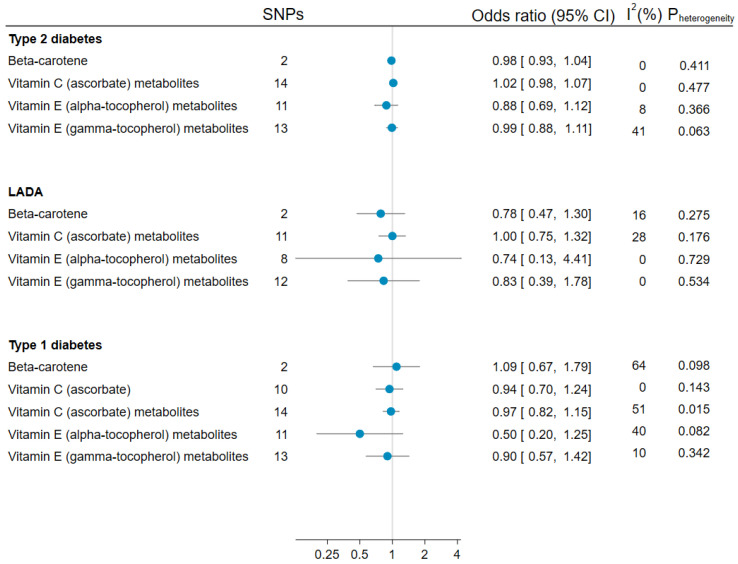
Summary ORs and 95% Cis for the effect of per unit increase in genetically predicted log_e_-transformed beta-carotene (μg/L), 1 μmol/L increase in ascorbate, and per unit increase in log_10_-transformed ascorbate, alpha-tocopherol, and gamma-tocopherol metabolite concentrations on LADA, type 1 diabetes, and type 2 diabetes. Estimates are based on the inverse-variance weighted method with multiplicative random effects.

Genetically predicted circulating beta-carotene, vitamin C (ascorbate), and vitamin E (alpha-tocopherol or gamma-tocopherol metabolites) were not significantly associated with the risk of LADA, type 1, or type 2 diabetes in MR analyses (Figure 2). For genetic predisposition to higher alpha-tocopherol levels, the ORs were estimated at 0.88 (95% CI 0.69, 1.12) for type 2 diabetes, 0.74 (95% CI 0.13, 4.41) for LADA, and 0.50 (95% CI 0.20, 1.25) for type 1 diabetes. Evidence of substantial heterogeneity was only seen for the associations between type 1 diabetes and beta-carotene or vitamin C (ascorbate) metabolites (Figure 2). This did not seem to be attributed to horizontal pleiotropy for vitamin C metabolites (Figure S13), while horizontal pleiotropy could not be assessed for beta-carotene.

### 3.6. Sensitivity Analyses

The inverse associations of vitamin C and vitamin E with LADA^high^ remained largely unaffected in sensitivity analyses (Figures S17 and S18). For type 2 diabetes, associations with beta-carotene and selenium were not significant in most sensitivity analyses (Figures S16 and S19) but estimates for zinc were minimally affected (Figure S20). Because only 29% of individuals with diabetes participated in the study within three months of diagnosis, associations were more uncertain after excluding those with longer diabetes duration. In stratified analyses by smoking status, the inverse associations of vitamin C and vitamin E with LADA^high^ were primarily seen among non-smokers (Figures S17 and S18). Stratifying LADA cases by HLA risk genotype did not indicate that associations between antioxidant intake and incident diabetes were modified by genetic susceptibility (Table S8).

## 4. Discussion

### 4.1. Main Findings

We investigated whether a high intake of antioxidant nutrients is linked to a lower risk of LADA and type 2 diabetes and examined the presence of causal relationships between circulating antioxidants and diabetes. We found that higher intakes of vitamin C and vitamin E were associated with a lower risk of LADA^high^, but not LADA^low^ or type 2 diabetes. No firm conclusions regarding the potential causality of these associations could be drawn based on the MR analyses. However, there was some indication that type 1 diabetes and LADA may be less likely in those with higher genetically predicted vitamin E, but not vitamin C levels. Evidence for a potential protective effect of higher vitamin E intake was supported by the observed association with better beta cell function and lower insulin resistance. There was no indication that a high intake of beta-carotene, selenium, and zinc would reduce the risk of either LADA or type 2 diabetes.

### 4.2. Findings in Relation to Previous Studies

To our knowledge, there is no prior publication regarding antioxidants and LADA, but there are some studies of autoimmune diabetes in children that show similar results. Specifically, our finding of an inverse association between vitamin C intake and risk of LADA^high^ is consistent with a meta-analysis of two case-control studies that found a 53% lower risk of childhood type 1 diabetes related to higher vitamin C intakes [5]. Nevertheless, our MR analyses of circulating ascorbate levels did not verify that vitamin C influences the development of either LADA or type 1 diabetes. This finding confirms the results of The Environmental Determinants of the Diabetes In the Young (TEDDY) study that found no association between plasma ascorbic acid levels and type 1 diabetes [25]. However, the TEDDY study revealed that those with higher plasma ascorbic acid levels had a lower risk of islet autoimmunity [25], suggesting that vitamin C may be protective against autoimmune diabetes at early stages but may not be related to disease progression. Furthermore, our estimate of an inverse association between dietary vitamin E and LADA^high^ is similar to that from the only previous study of type 1 diabetes, which, however, had a small sample size and, accordingly, limited statistical power to identify significant associations [26]. In addition, two prospective studies of type 1 diabetes in children and adults, respectively, suggest a protective role of high serum alpha-tocopherol levels [27,28]. LADA^high^ is the group that resembles type 1 diabetes the most in terms of the degree of autoimmunity, presence of HLA risk genotypes, and insulin deficiency. This could explain why associations with vitamin C and vitamin E were only seen among LADA^high^, but not LADA^low^, which is more similar to type 2 diabetes. Notably, these associations were primarily seen among non-smokers. This is not surprising, considering that smoking causes oxidative stress and is linked to reductions in plasma antioxidants, including vitamin C [29] and vitamin E [30]. Accordingly, smoking may neutralize the potentially beneficial effects of vitamin C and vitamin E on the incidence of autoimmune diabetes. Other factors associated with oxidative stress, including radiation, psychological stress, and physical exercise [31], may also modify the associations between antioxidants and LADA. Our MR point estimates for the association between genetically predicted alpha-tocopherol levels, as an indicator of vitamin E exposure, and LADA or type 1 diabetes were compatible with reduced risk, but not statistically significant. At the same time, estimates for the association with gamma-tocopherol levels were closer to 1, which may be explained by its lower antioxidant reactivity towards free radicals compared to alpha-tocopherol [32]. Our observation that intakes of vitamin C and vitamin E were not associated with type 2 diabetes, either in the observational or MR analyses, is in line with previous MR studies [11,33] and randomized controlled trials [34,35,36].

Biological mechanisms linking vitamin C and vitamin E intakes to the risk of LADA, may involve the essential role of these antioxidants in inhibiting lipid peroxidation. This process refers to a chain reaction initiated by free radicals, which leads to the oxidation of phospholipids in the cellular membranes, and has been linked to beta cell dysfunction [37]. Both vitamin C and vitamin E are known free radical scavengers with distinctive features. On the one hand, vitamin E is an efficient lipophilic antioxidant with the ability to act as a chain-breaker of lipid peroxidation, preventing the proliferation of free radicals in cell membranes [38]. On the other hand, vitamin C is an important hydrophilic antioxidant with the ability to neutralize free radicals by donating an electron, as well as to restore the antioxidant function of vitamin E [39]. This does not necessarily reinforce a recommendation to take antioxidant supplements, especially high doses of individual antioxidants. In fact, a randomized control trial of healthy adults failed to identify any further effects of vitamin E supplementation on lipid peroxidation [40]. In contrast, a diet rich in vitamin C and vitamin E, including fruits, vegetables, nuts, and olive oil may be beneficial [41,42]. Besides preventing oxidative stress, such a diet may enhance the gut microbiome, which is also thought to play a role in the development of both autoimmune [43] and type 2 diabetes [44]. Furthermore, our findings did not suggest any protective effects of receiving individual antioxidant supplements on diabetes incidence. However, we found an inverse relationship between the intake of multivitamins and both LADA and type 2 diabetes, which could reflect the previously described synergistic effects between vitamin C and vitamin E in inhibiting lipid peroxidation [45]. This observation may also reflect inverse associations with other vitamins. For instance, vitamin D is thought to be beneficial for the prevention of autoimmune diabetes, primarily due to its immunoregulatory function [46]. Indeed, observational studies of vitamin D supplementation suggest inverse associations with both type 1 diabetes [5] and LADA [47]. We additionally confirmed previous findings of an inverse association between vitamin E and insulin resistance [48,49] and identified a positive association with beta cell function, particularly among individuals with LADA, which provides some support that a causal relationship between vitamin E intake and autoimmune diabetes may exist. Interestingly, vitamin E was not associated with insulin resistance in LADA patients, indicating that factors other than oxidative stress (e.g., inflammation) may play a primary role in inducing insulin resistance. We found no indication of a protective effect of beta-carotene, selenium, or zinc on LADA. In line with that, previous studies in children did not observe an association between serum beta-carotene levels and islet autoimmunity [50], or between type 1 diabetes and intake [26] or circulating levels [51] of zinc, whereas selenium has not been previously investigated in relation to autoimmune diabetes.

There was some indication that a high intake of beta-carotene may increase the risk of type 2 diabetes and LADA^low^, but previous observational studies [52], MR analyses [33] including ours, and clinical trials [35,36] do not indicate adverse effects on type 2 diabetes. We also observed an increased risk of type 2 diabetes in relation to a high intake of zinc in line with some [53,54], but not all [10], previous observational studies. Importantly, there is no support from randomized controlled trials [55] or MR studies [33] that intakes of zinc have a causal effect on type 2 diabetes incidence. Excess risk of type 2 diabetes was also observed in relation to a high selenium intake, and this finding is supported by previous observational [56], experimental [57], and MR studies [58]. It has been hypothesized that a plausible mechanism could be through promoting insulin resistance. Specifically, evidence from animal studies has shown that excess production of glutathione peroxidase 1, which is the most common type of selenoprotein, may disrupt the insulin signalling pathway and lead to insulin resistance [59,60].

### 4.3. Strengths and Limitations

This is the first study to investigate antioxidant nutrient intake and LADA and to perform MR analyses on the association between antioxidant nutrients and LADA or type 1 diabetes. Its major strength is the triangulation of evidence using both an observational study design and an MR approach. Additionally, observational data came from a population-based study with more than 500 individuals with LADA with detailed information on diet, lifestyle, and genetic and clinical characteristics. Accordingly, we had the possibility to account for a wide range of confounders and to examine potential mechanisms for the observed associations.

An important limitation of this study is the use of self-reported dietary information and the retrospective design. Relying on memory to recollect dietary habits can be challenging, particularly among cases who were asked about their diet before diagnosis. It seems possible that those afflicted with diabetes have increased their consumption of antioxidant-rich foods, following medical advice, and may have, thus, overreported their exposure before diagnosis. This would lead to an underestimation of the observed associations between antioxidants and diabetes. Nevertheless, cases were invited to ESTRID soon after diabetes diagnosis and carefully instructed to report their diet as it was prior to diagnosis, using a validated FFQ. Additionally, sensitivity analyses excluding individuals with a diabetes duration > 3 months or who changed their diet after diagnosis had a small influence on the results. Another limitation is the lack of data on the frequency and quantity of antioxidant nutrient supplementation, as this information may increase the overall validity of FFQ-derived nutrient estimates and help classify them correctly into categories [21]. However, few individuals reported using supplements in our study, and most of those who did were using multivitamins. Because individuals who use micronutrient supplements likely have lower micronutrient intakes from diet, in sensitivity analyses we excluded those that reported using antioxidant supplements, but this did not influence the observed associations. While our study included GWAS data on type 1 diabetes, in addition to LADA and type 2 diabetes, we did not have access to observational data on this outcome. This limited our ability to compare associations with antioxidant nutrient intakes between the two autoimmune diabetes forms, LADA and type 1 diabetes. A limitation of the MR design is that the validity of genetic instruments relies on assumptions that cannot fully be tested. Specifically, estimates can be biased when horizontal pleiotropy is present, i.e., associations between the genetic instruments and diabetes through pathways other than antioxidant nutrients, which may have introduced genetic confounding. Nevertheless, MR-Egger did not suggest the presence of horizontal pleiotropy in our estimates, and SNPs with known pleiotropic effects were excluded from our analyses. Notably, only a small fraction of the variance in circulating antioxidants, particularly vitamin E (alpha-tocopherol) metabolites (3.3%) and vitamin C (ascorbate) (1.9%), was explained by the SNPs used. This limitation, in addition to the small GWAS of LADA and type 1 diabetes, in contrast to the large-scale GWAS meta-analysis of type 2 diabetes, resulted in low statistical power to detect causal relationships with genetically predicted antioxidants. Additionally, it was not possible to perform MR analyses separately for LADA^high^ and LADA^low^, since such GWAS is not currently available. Finally, our findings are based on European populations, and generalizability to continents with entirely different diets or genetic makeup may be limited.

## 5. Conclusions

This study suggests that vitamin E may play a protective role in the incidence of LADA, potentially by inhibiting the development of autoimmunity and preventing insulin resistance. A diet rich in vitamin E, including plant-based oils, nuts, seeds, and dark-green leafy vegetables, is generally considered part of a healthy lifestyle and should be encouraged as a public health strategy. Additionally, our study supports previous findings of an adverse effect of selenium on type 2 diabetes. Further studies are certainly needed to confirm our findings and clarify the potential mechanisms for the observed associations.

## Figures and Tables

**Table 1:** Characteristics of ESTRID participants.

	Controls	Type 2 Diabetes	LADA	*p*-value ^1^	LADA^low 2^	LADA^high 2^	*p*-value ^3^
*n*	2276	1989	584		262	313	
Age (years), mean (SD)	59.0 (13.8)	63.3 (10.4)	59.1 (12.4)	<0.001	60 (12.1)	58.3 (12.5)	0.100
BMI, mean (SD)	26.0 (4.2)	31.2 (5.4)	28.5 (5.7)	<0.001	29.4 (6.0)	27.7 (5.3)	<0.001
Women, %	52.6	39.9	47.3	0.002	43.1	51.1	0.056
University education, %	37.7	20.4	28.3	<0.001	27.1	29.7	0.033
Physically inactive, %	14.1	25.1	18.3	<0.001	21.8	16.0	0.162
Current smoking, %	18.5	21.6	23.0	<0.001	17.9	26.2	0.029
Low alcohol intake, %	33.3	36.6	37.7	0.005	39.3	36.4	0.437
High-risk HLA ^4^, %	-	31.4	60.3	<0.001	52.4	67.7	<0.001
Family history of diabetes, %	25.4	51.0	45.4	0.016	45.0	44.7	0.941
CVD or hypertension history, %	26.8	58.9	42.3	<0.001	45.4	39.9	0.408
Insulin treatment, %	-	5.6	32.1	<0.001	23.0	39.6	0.001
GADA (U/mL), median (IQR)	-	-	250 (32, 250)	-	28.5 (17, 73)	250 (250, 250)	<0.001
HOMA-B ^5^, median (IQR)	-	71.2 (44.4, 96.0)	40.5 (14.6, 70.0)	<0.001	50.6 (22.0, 83.6)	29.5 (12, 59)	<0.001
HOMA-IR ^5^, median (IQR)	-	3.6 (2.7, 4.8)	2.8 (1.8, 4.4)	<0.001	3.2 (2.2, 4.9)	2.4 (1.7, 4.0)	<0.001

^1^ *p* for the comparison between individuals with LADA and type 2 diabetes. ^2^ Nine individuals with LADA did not consent to have information on their GADA levels accessed. ^3^ *p* for the comparison between LADA^low^ and LADA^high^ individuals. ^4^ Available for 64% of cases (LADA *n* = 392, type 2 diabetes *n* = 1251). ^5^ Available for 85% of cases (LADA *n* = 471, type 2 diabetes *n* = 1713). CVD: Cardiovascular diseases.

**Table 2:** ORs and 95% Cis of type 2 diabetes, LADA, LADA^low^, and LADA^high^ in relation to antioxidant nutrient intakes.

Antioxidant Nutrient Intake ^1^	Type 2 Diabetes		LADA		LADA^low^		LADA^high^	
	N Cases/Controls	OR (95% CI)	N Cases/Controls	OR (95% CI)	N Cases/Controls	OR (95% CI)	N Cases/Controls	OR (95% CI)
Beta-carotene (µg/day)								
Q1 (<1642.4)	556/569	1.00	156/569	1.00	67/569	1.00	85/569	1.00
Q2 (1642.4 to < 2595.6)	422/569	0.91 (0.73, 1.13)	151/569	1.15 (0.87, 1.51)	55/569	1.05 (0.70, 1.57)	96/569	1.21 (0.86, 1.70)
Q3 (2595.6 to < 4072.1)	428/569	0.89 (0.71, 1.11)	121/569	0.86 (0.64, 1.14)	56/569	1.01 (0.67, 1.51)	62/569	0.73 (0.50, 1.06)
Q4 (≥4072.1)	583/569	1.15 (0.92, 1.43)	156/569	1.16 (0.87, 1.54)	84/569	1.62 (1.10, 2.40)	70/569	0.87 (0.60, 1.26)
Per 1 SD (2670)		1.09 (1.01, 1.17)		1.04 (0.94, 1.15)		1.19 (1.05, 1.35)		0.88 (0.75, 1.03)
Supplementation (ever vs. never)	36/52	1.11 (0.63, 1.94)	9/52	0.76 (0.36, 1.60)	3/52	0.60 (0.18, 2.06)	6/52	0.85 (0.35, 2.06)
Vitamin C (mg/day)								
Q1 (<73.6)	537/569	1.00	174/569	1.00	72/569	1.00	99/569	1.00
Q2 (73.6 to < 104.2)	487/569	0.93 (0.75, 1.15)	158/569	0.96 (0.74, 1.25)	66/569	0.97 (0.66, 1.43)	88/569	0.92 (0.66, 1.28)
Q3 (104.2 to < 144.6)	439/569	0.82 (0.65, 1.02)	138/569	0.79 (0.60, 1.04)	65/569	0.90 (0.61, 1.33)	73/569	0.70 (0.50, 0.99)
Q4 (≥144.6)	526/569	0.99 (0.80, 1.24)	114/569	0.66 (0.49, 0.88)	59/569	0.80 (0.54, 1.20)	53/569	0.51 (0.35, 0.76)
Per 1 SD (64.2)		1.05 (0.98, 1.13)		0.91 (0.82, 1.01)		0.97 (0.84, 1.12)		0.84 (0.73, 0.98)
Supplementation (ever vs. never)	180/268	0.85 (0.65, 1.10)	65/268	1.00 (0.73, 1.36)	29/268	1.01 (0.65, 1.58)	35/268	0.96 (0.65, 1.43)
Vitamin E (mg/day)								
Q1 (<7.2)	467/569	1.00	159/569	1.00	63/569	1.00	92/569	1.00
Q2 (7.2 to < 8.7)	393/569	0.87 (0.69, 1.09)	158/569	1.01 (0.78, 1.33)	63/569	0.94 (0.63, 1.40)	94/569	1.09 (0.79, 1.52)
Q3 (8.7 to < 10.6)	497/569	0.96 (0.77, 1.20)	123/569	0.79 (0.59, 1.05)	55/569	0.86 (0.57, 1.30)	66/569	0.75 (0.53, 1.08)
Q4 (≥10.6)	632/569	1.03 (0.82, 1.30)	144/569	0.84 (0.63, 1.13)	81/569	1.12 (0.75, 1.69)	61/569	0.66 (0.45, 0.97)
Per 1 SD (3.0)		1.03 (0.95, 1.12)		0.93 (0.84, 1.04)		1.08 (0.94, 1.25)		0.80 (0.69, 0.94)
Supplementation (ever vs. never)	67/81	1.05 (0.67, 1.63)	19/81	0.91 (0.53, 1.57)	9/81	0.89 (0.42, 1.91)	10/81	0.94 (0.47, 1.88)
Selenium (µg/day)								
Q1 (<33.4)	375/569	1.00	130/569	1.00	53/569	1.00	76/569	1.00
Q2 (33.4 to < 40.2)	422/569	1.03 (0.81, 1.30)	134/569	0.97 (0.73, 1.29)	55/569	0.92 (0.60, 1.40)	76/569	0.98 (0.69, 1.41)
Q3 (40.2 to < 48.7)	512/569	1.11 (0.88, 1.41)	159/569	1.10 (0.82, 1.46)	71/569	1.10 (0.73, 1.67)	86/569	1.05 (0.73, 1.50)
Q4 (≥48.7)	680/569	1.29 (1.01, 1.64)	161/569	1.10 (0.81, 1.49)	83/569	1.26 (0.82, 1.93)	75/569	0.93 (0.63, 1.37)
Per 1 SD (13.5)		1.08 (1.00, 1.18)		1.00 (0.90, 1.11)		1.05 (0.91, 1.21)		0.94 (0.81, 1.08)
Supplementation (ever vs. never)	48/81	0.91 (0.57, 1.45)	18/81	1.03 (0.59, 1.79)	11/81	1.48 (0.73, 3.01)	6/81	0.60 (0.25, 1.42)
Zinc (mg/day)								
Q1 (<8.6)	325/569	1.00	116/569	1.00	42/569	1.00	71/569	1.00
Q2 (8.6 to < 9.8)	420/569	1.28 (1.01, 1.63)	147/569	1.24 (0.93, 1.65)	67/569	1.41 (0.92, 2.16)	79/569	1.15 (0.80, 1.65)
Q3 (9.8 to < 11.5)	515/569	1.27 (1.00, 1.61)	166/569	1.19 (0.88, 1.60)	71/569	1.15 (0.74, 1.80)	93/569	1.26 (0.87, 1.83)
Q4 (≥11.5)	729/569	1.34 (1.03, 1.74)	155/569	1.00 (0.71, 1.40)	82/569	1.17 (0.72, 1.91)	70/569	0.88 (0.57, 1.37)
Per 1 SD (2.5)		1.15 (1.06, 1.25)		1.03 (0.92, 1.15)		1.09 (0.93, 1.27)		0.98 (0.84, 1.14)
Supplementation (ever vs. never)	61/92	1.01 (0.67, 1.54)	23/92	1.05 (0.64, 1.73)	9/92	0.93 (0.43, 1.98)	13/92	0.96 (0.52, 1.79)
Multivitamins (ever vs. never)	284/519	0.76 (0.61, 0.93)	93/519	0.71 (0.54, 0.92)	41/519	0.74 (0.51, 1.08)	50/519	0.66 (0.47, 0.92)

^1^ Adjusted for energy intake except for supplementation. ORs adjusted for age, sex, education, physical activity, smoking, intake of alcohol, family history of diabetes, BMI, tertiles of fatty fish (except for selenium), red meat (except for selenium and zinc), sweetened beverages, and coffee intake, and history of cardiovascular diseases or hypertension.

**Table 3:** Beta-coefficients of log_e_-transformed HOMA-B and HOMA-IR in relation to 1 SD higher antioxidant nutrient intakes among individuals with type 2 diabetes or LADA.

Antioxidant Nutrient Intake (Per 1 SD) ^1^	HOMA-B			HOMA-IR		
	All	Type 2 Diabetes	LADA	All	Type 2 Diabetes	LADA
Beta-carotene (2670 µg/day)	0.017 (−0.009, 0.043)	0.009 (−0.017, 0.034)	0.018 (−0.050, 0.086)	−0.021 (−0.046, 0.004)	−0.015 (−0.041, 0.011)	−0.051 (−0.118, 0.017)
Vitamin C (64.2 mg/day)	0.013 (−0.013, 0.038)	0.002 (−0.022, 0.026)	0.007 (−0.069, 0.083)	−0.002 (−0.027, 0.022)	−0.009 (−0.034, 0.016)	0.007 (−0.069, 0.083)
Vitamin E (3.0 mg/day)	0.043 (0.015, 0.072)	0.027 (0.000, 0.054)	0.100 (0.021, 0.179)	−0.042 (−0.069, −0.015)	−0.037 (−0.065, −0.009)	−0.077 (−0.156, 0.003)
Selenium (13.5 µg/day)	0.010 (−0.020, 0.040)	0.008 (−0.021, 0.036)	0.022 (−0.066, 0.111)	−0.014 (−0.043, 0.014)	−0.003 (−0.033, 0.026)	−0.082 (−0.169, 0.005)
Zinc (2.5 mg/day)	0.018 (−0.010, 0.046)	0.011 (−0.015, 0.037)	0.048 (−0.038, 0.134)	−0.019 (−0.046, 0.007)	−0.021 (−0.048, 0.006)	−0.022 (−0.107, 0.064)

^1^ Adjusted for energy intake. Beta-coefficients adjusted for age, sex, education, physical activity, smoking, intake of alcohol, family history of diabetes, BMI, tertiles of fatty fish (except for selenium), red meat (except for selenium and zinc), sweetened beverages, and coffee intake, and history of cardiovascular diseases or hypertension.

## Data Availability

The datasets described in the manuscript and the analytic code are available from the corresponding author upon reasonable request. MR analyses were based on publicly available summary statistics.

## Supplementary Materials

The following supporting information can be downloaded at: https://www.mdpi.com/article/10.3390/nu15112546/s1, Table S1: Instrumental variables and their sources; Table S2: Characteristics of ESTRID participants across quartiles of beta-carotene intakes; Table S3: Characteristics of ESTRID participants across quartiles of vitamin C intakes; Table S4: Characteristics of ESTRID participants across quartiles of vitamin E intakes; Table S5: Characteristics of ESTRID participants across quartiles of selenium intakes; Table S6: Characteristics of ESTRID participants across quartiles of zinc intakes; Table S7: ORs and 95% cIs of LADA in relation to antioxidant nutrient intakes; Table S8: ORs and 95% cIs of LADA with low/intermediate- or high-risk HLA genotype in relation to antioxidant nutrient intakes; Table S9: ORs and 95% cIs of type 2 diabetes in relation to antioxidant nutrient intakes; Figure S1: ESTRID case-control design and flow-diagram of study participants; Figure S2: Flow-diagram of two-sample Mendelian randomization analyses; Figures S3–S6: Two-sample Mendelian randomization analysis on the association between genetically predicted circulating antioxidants and type 2 diabetes; Figures S7–S10: Two-sample Mendelian randomization analysis on the association between genetically predicted circulating antioxidants and LADA; Figures S11–S15: Two-sample Mendelian randomization analysis on the association between genetically predicted circulating antioxidants and type 1 diabetes; Figure S16: ORs and 95% cIs of type 2 diabetes, LADA, LADA^low^, and LADA^high^ in relation to 1 SD higher beta-carotene intakes, in different sensitivity analyses; Figure S17: ORs and 95% cIs of type 2 diabetes, LADA, LADA^low^, and LADA^high^ in relation to 1 SD higher vitamin C intakes, in different sensitivity analyses; Figure S18: ORs and 95% cIs of type 2 diabetes, LADA, LADA^low^, and LADA^high^ in relation to 1 SD higher vitamin E intakes, in different sensitivity analyses; Figure S19: ORs and 95% cIs of type 2 diabetes, LADA, LADA^low^, and LADA^high^ in relation to 1 SD higher selenium intakes, in different sensitivity analyses; Figure S20: ORs and 95% cIs of type 2 diabetes, LADA, LADA^low^, and LADA^high^ in relation to 1 SD higher zinc intakes, in different sensitivity analyses.

## Appendix A

### Appendix A.1. Genetic Instruments for Circulating Antioxidants

Three single nucleotide polymorphisms (SNPs) associated with plasma levels of beta-carotene (*p* < 5 × 10^−8^) were obtained from a GWAS of 2344 women in the Nurses’ Health Study [61]. Two SNPs were in linkage disequilibrium (R^2^  ≥  0.01), and the one with the largest *p*-value (rs12934922) was excluded. With regard to vitamin C, 11 SNPs strongly associated with plasma concentrations of ascorbate (*p* < 5 × 10^−8^) were derived from a genome-wide meta-analysis including 52,018 individuals; however, 1 SNP (rs174547) with pleiotropic effect, i.e., associated also with glycerophospholipid and sphingolipid concentrations [11], was excluded from the MR analyses. Summary statistics for the association with the remaining 10 SNPs were only identified in the GWAS of type 1 diabetes, but not of LADA or type 2 diabetes. Three SNPs strongly associated with circulating alpha-tocopherol levels were identified in a GWAS of 5005 men [62], but those have also been related to lipid metabolism and/or regulation, and thus were not included in the MR analyses. Loci associated with circulating metabolites of ascorbate (14 SNPs), alpha-tocopherol (11 SNPs), and gamma-tocopherol (13 SNPs) at suggestive genome-wide significance level (*p* < 1 × 10^−5^) were derived from a GWAS with 7824 adults [63]. Three SNPs associated with ascorbate (rs6834631, rs8057559, rs6826474) or alpha-tocopherol (rs10163969, rs10245705, rs7930821) metabolites and one SNP associated with gamma-tocopherol metabolites (rs10520845) were unavailable in the GWAS of LADA and thus were not included in some of the MR analyses.

### Appendix A.2. Statistical Analysis

Genetic instruments with an F-statistic >10 were considered strong enough to be included in MR analyses. F-statistic was calculated based on the proportion of the exposure variance explained by the genetic variants (R^2^), the number of instruments (K), and the sample size (N), as $F=\left(\frac{N-K-1}{K}\right)\left(\frac{R^{2}}{1-R^{2}}\right)$ [64]. The proportion of the exposure variance explained by the genetic variants ranged from 1.9% to 18.6% (Table S1). Wald ratios were estimated for each SNP and correspond to the odds ratio of LADA, type 1 diabetes, or type 2 diabetes per unit increase in circulating antioxidants or their metabolites. In primary analyses, Wald ratios were pooled using the multiplicative random-effects inverse-variance weighted method, which is considered the most efficient method when directional pleiotropy is absent and instrumental variables are valid [65]. Sensitivity analyses were performed when ≥ 3 SNPs were available, and included the MR-Egger regression, which can identify and correct for pleiotropic effects but is sensitive to outliers and may provide imprecise estimates [66]. The presence of horizontal pleiotropy was assessed with the *p*-value for the intercept in the MR-Egger regression [66]. We also used the weighted median- [67] and mode-based methods [68], which are less sensitive to outliers and assume that the majority or plurality, respectively, of the genetic variants are valid instruments.
