# Supplementary material for: Antioxidant Nutrients and Risk of Latent Autoimmune Diabetes in Adults and Type 2 Diabetes: A Swedish Case-Control Study and Mendelian Randomization Analysis
Source: Nutrients. 2023 May 30;15(11):2546. doi: 10.3390/nu15112546 (PMC10255589; doi:10.3390/nu15112546)
Supplement: Supplementary file 1 [file nutrients-15-02546-s001.zip › nutrients-2386376-supplementary.pdf]

SUPPLEMENTARY MATERIAL

Antioxidant nutrients and risk of latent autoimmune diabetes in adults and type 2 diabetes: a Swedish case-control study and Mendelian randomization analysis

Anna-Maria Lampousi[1], Josefin E. Löfvenborg[1,2], Emma Ahlqvist[3], Tiinamaija Tuomi[3,4,5,6], Alicja Wolk[1], and Sofia Carlsson[1]

[1]Institute of Environmental Medicine, Karolinska Institute, Stockholm, Sweden
[2]Department of Risk and Benefit Assessment, Swedish Food Agency, Uppsala, Sweden
[3]Department of Clinical Sciences, Lund University, Malmö, Sweden
[4]Institute for Molecular Medicine Finland (FIMM) and Research Programs Unit, Clinical and Molecular Metabolism, University of Helsinki, Helsinki, Finland
[5]Department of Endocrinology, Helsinki University Hospital, Helsinki, Finland
[6]Folkhälsan Research Center, Helsinki, Finland

Correspondence to:
Anna-Maria Lampousi
Institute of Environmental Medicine, Karolinska Institutet
Box 210, 171 77 Stockholm, Sweden
+46702575629
annamaria.lampousi@ki.se

Table S1. Instrumental variables and their sources

Antioxidant	SNPs	N	Unit	Variance	PMID
**Beta-carotene**					
Absolute	2	2,344	μg/L in log_e-transformed scale	9.0%	23134893
**Ascorbate**					
Absolute	10	52,018	μmol/L	1.9%	33203707
Metabolites	14	2,063	log_{10}-transformed	18.6%	24816252
**Alpha-tocopherol**					
Absolute*	3	5,006	mg/L in log_{10}-transformed scale	1.7%	21729881
Metabolites	11	7,276	log_{10}-transformed	3.3%	24816252
**Gamma-tocopherol**					
Metabolites	13	5,822	log_{10}-transformed	15.0%	24816252

* Not included in Mendelian randomization analyses

Table S2. Characteristics of ESTRID participants across quartiles of beta-carotene intakes. Values are presented as mean (SD), unless otherwise specified.

	**Beta-carotene**			
	Q1 (<1642.4 µg/day)	*Q2 (1642.4 to <2595.6 µg/day)*	*Q3 (2595.6 to <4,072.1 µg/day)*	*Q4 (≥ 4072.1 µg/day)*
n	1,281	1,142	1,118	1,308
Age (years)	59.4 (12.9)	60.5 (12.6)	60.6 (12.5)	62.5 (11.9)
BMI	29.0 (5.8)	28.1 (5.1)	28.0 (4.9)	28.5 (5.7)
Women, %	34.4	46.2	49.2	57.2
University education, %	25.4	32.0	31.6	29.4
Physically inactive, %	24.9	20.5	16.1	14.8
Current smoking, %	22.9	20.2	17.1	16.7
Family history of diabetes, %	36.1	35.1	39.5	42.1
History of CVD or hypertension, %	39.7	39.1	40.9	47.0
Fruit (g/day)	127.9 (116.9)	154.4 (125.6)	161.4 (123.9)	173.5 (129.7)
Vegetables (g/day)	134.5 (96.1)	194.2 (113.4)	230 (133.9)	282.6 (167.7)
Nuts (g/day)	3.4 (5.3)	4.1 (5.7)	4.0 (5.8)	4.1 (6.5)
Fish (g/day)	43.5 (36.2)	47.2 (33.4)	47.8 (40.7)	44.7 (30.6)
Fatty fish (g/day)	20.3 (21.6)	21.7 (18.6)	22.1 (20.5)	20.8 (17.8)
Red and processed meat (g/day)	110.7 (65.7)	96.1 (54.6)	89.6 (53.9)	74.0 (44.6)
Dairy products (g/day)	549.8 (433.5)	425.1 (314)	351. 7 (264.0)	289.4 (222.8)
Energy adjusted whole grain (g/day)	41.0 (31.4)	49.9 (31.8)	57.4 (33.0)	69.0 (47.7)
Sweetened beverages (g/day)	194.4 (413.3)	94.1 (219.1)	64.7 (151.9)	42.5 (130.9)
Coffee (g/day)	465.1 (355.6)	442.0 (334.0)	438.1 (335.3)	422.1 (320.0)
Energy adjusted alcohol (g/day)	7.6 (9.8)	9.1 (10.1)	9.3 (10.4)	9.1 (11.5)
Olive oil, %	35.2	41.9	47.0	42.5
Corn or sunflower oil, %	13.8	13.0	12.5	11.6
Rapeseed oil, %	37.0	49.4	48.8	50.9

CVD: Cardiovascular diseases

Table S3. Characteristics of ESTRID participants across quartiles of vitamin C intakes. Values are presented as mean (SD), unless otherwise specified.

	**Vitamin C**			
	Q1 (<73.6 mg/day)	*Q2 (73.6 to <104.2 mg/day)*	*Q3 (104.2 to <144.6 mg/day)*	*Q4 (≥144.6 mg/day)*
n	1,280	1,214	1,146	1,209
Age (years)	59.3 (13.2)	61.7 (12.8)	61.0 (12.1)	61.1 (11.6)
BMI	28.7 (5.7)	28.2 (5.5)	28.2 (5.3)	28.5 (5.5)
Women, %	39.7	49.4	48.8	49.6
University education, %	25.8	29.6	33.9	34.2
Physically inactive, %	25.0	18.5	17.2	15.4
Current smoking, %	23.8	19.2	16.8	17.0
Family history of diabetes, %	37.0	38.7	38.7	38.9
History of CVD or hypertension, %	38.6	41.8	43.5	43.8
Fruit (g/day)	98.5 (80.7)	144.2 (105.2)	169.8 (124.1)	208.0 (154.2)
Vegetables (g/day)	148.4 (97.6)	193.1 (106.4)	231.2 (146.4)	275.2 (174.9)
Nuts (g/day)	3.7 (5.9)	4.2 (6.1)	3.9 (6.0)	3.7 (5.4)
Fish (g/day)	46.3 (39.0)	46.1 (30.0)	47.4 (39.3)	43.0 (31.9)
Fatty fish (g/day)	21.3 (22.8)	21.4 (17.4)	21.8 (19.9)	20.3 (18.0)
Red and processed meat (g/day)	112.8 (68.7)	94.9 (52.2)	88.5 (49.9)	72.3 (44.8)
Dairy products (g/day)	575.1 (422.6)	427.1 (297.4)	344.6 (255.1)	258.4 (204.0)
Whole grain (g/day)	42.3 (28.1)	50.2 (31.2)	55.0 (35.2)	71.1 (49.8)
Sweetened beverages (g/day)	143.1 (322.4)	98.3 (225.4)	92.0 (303.1)	63.4 (170.7)
Coffee (g/day)	496.5 (385.9)	449.5 (318.4)	417.4 (296.6)	399 (326.6)
Alcohol (g/day)	7.4 (9.3)	8.3 (9.4)	9.9 (11.6)	9.6 (11.4)
Olive oil, %	36.3	39.0	43.9	47.2
Corn or sunflower oil, %	12.3	13.1	13.0	12.7
Rapeseed oil, %	43.1	47.2	45.7	49.7

CVD: Cardiovascular diseases

Table S4. Characteristics of ESTRID participants across quartiles of vitamin E intakes. Values are presented as mean (SD), unless otherwise specified.

	**Vitamin E**			
	Q1 (<7.2 mg/day)	*Q2 (7.2 to <8.7 mg/day)*	*Q3 (8.7 to <10.6 mg/day)*	*Q4 (≥10.6 mg/day)*
n	1,195	1,120	1,189	1,345
Age (years)	59.6 (13.0)	60.3 (12.6)	61.3 (12.2)	61.7 (12.2)
BMI	28.5 (5.9)	28.0 (5.4)	28.4 (5.5)	28.7 (5.3)
Women, %	58.4	55.3	45.6	30.3
University education, %	29.0	29.4	32.1	27.5
Physically inactive, %	22.4	18.7	15.8	19.5
Current smoking, %	22.5	18.8	17.8	18.1
Family history of diabetes, %	34.9	38.8	38.6	40.6
History of CVD or hypertension, %	38.8	39.2	42.1	46.4
Fruit (g/day)	154.2 (125.1)	160.2 (121.0)	153.7 (119.1)	149.8 (133.8)
Vegetables (g/day)	186.9 (125.8)	212.5 (139.0)	216.7 (143.5)	225.5 (155.0)
Nuts (g/day)	3.2 (4.6)	3.8 (5.1)	4.2 (6.3)	4.3 (6.9)
Fish (g/day)	45.1 (37.3)	45.0 (30.4)	45.3 (31.2)	47.2 (40.3)
Fatty fish (g/day)	20.1 (20.3)	20.6 (17.0)	21.1 (18.5)	22.8 (22.0)
Red and processed meat (g/day)	107.3 (68.1)	93.8 (53.4)	90.5 (51.2)	80.0 (49.9)
Dairy products (g/day)	658.6 (445.9)	420.9 (255.3)	333.2 (232.8)	228.1 (180.8)
Whole grain (g/day)	36.8 (23.0)	47.9 (25.4)	56.1 (33.5)	74.2 (51.2)
Sweetened beverages (g/day)	196.1 (423.3)	85.3 (195.0)	67.2 (164.1)	55.4 (161.0)
Coffee (g/day)	465.7 (362.3)	462.7 (325.0)	420.5 (311.2)	422.0 (342.5)
Alcohol (g/day)	6.1 (8.5)	7.8 (8.9)	9.7 (11.4)	11.0 (11.9)
Olive oil, %	37.0	40.7	44.5	43.4
Corn or sunflower oil, %	13.2	11.4	14.3	12.0
Rapeseed oil, %	43.8	47.1	47.9	46.8

CVD: Cardiovascular diseases

Table S5. Characteristics of ESTRID participants across quartiles of selenium intakes. Values are presented as mean (SD), unless otherwise specified.

	**Selenium**			
	Q1 (<33.4 μg/day)	*Q2 (33.4 to <40.2 μg/day)*	*Q3 (40.2 to <48.7 μg/day)*	*Q4 (≥48.7 μg/day)*
n	1,074	1,125	1,240	1,410
Age (years)	60.6 (13.3)	60.0 (13.0)	61.0 (12.2)	61.3 (11.7)
BMI	27.8 (5.6)	28.1 (5.9)	28.5 (13.4)	29.0 (5.2)
Women, %	58.4	55.3	45.6	30.3
University education, %	29.0	29.4	32.1	27.5
Physically inactive, %	22.4	18.7	15.8	19.5
Current smoking, %	22.5	18.8	17.8	18.1
Family history of diabetes, %	34.9	38.8	38.6	40.6
History of CVD or hypertension, %	38.8	39.2	42.1	46.4
Fruit (g/day)	180.6 (150.0)	155.6 (117.1)	151.4 (121.4)	135.5 (109.7)
Vegetables (g/day)	195.0 (147.4)	208.2 (148.9)	216.8 (133.8)	219.7 (139.7)
Nuts (g/day)	5.1 (7.6)	3.8 (5.3)	3.7 (5.5)	3.2 (4.9)
Fish (g/day)	22.8 (14.3)	34.4 (16.4)	46.7 (20.9)	70.9 (48.8)
Fatty fish (g/day)	10.4 (8.2)	15.5 (10.2)	22.0 (13.4)	33.1 (27.9)
Red and processed meat (g/day)	72.3 (50.0)	82.0 (45.5)	99.4 (57.1)	110.0 (62.8)
Dairy products (g/day)	328.5 (242.1)	395.8 (302.4)	446.5 (351.2)	432.8 (391.6)
Whole grain (g/day)	49.6 (35.9)	52.7 (38.5)	53.5 (35.4)	60.3 (41.9)
Sweetened beverages (g/day)	150.0 (385.1)	98.7 (253.4)	94.7 (219.0)	67.4 (176.5)
Coffee (g/day)	425.5 (319.7)	439.3 (333.0)	452.7 (332.7)	446.9 (355.5)
Alcohol (g/day)	6.3 (8.4)	7.6 (9.5)	9.0 (10.3)	11.4 (12.1)
Olive oil, %	37.6	38.4	43.5	45.0
Corn or sunflower oil, %	12.1	12.0	13.2	13.4
Rapeseed oil, %	42.1	45.5	48.2	48.8

CVD: Cardiovascular diseases

Table S6. Characteristics of ESTRID participants across quartiles of zinc intakes. Values are presented as mean (SD), unless otherwise specified.

	**Zinc**			
	Q1 (<8.6 mg/day)	*Q2 (8.6 to <9.8 mg/day)*	*Q3 (9.8 to <11.5 mg/day)*	*Q4 (≥11.5 mg/day)*
n	1,010	1,136	1,250	1,453
Age (years)	59.9 (13,3)	61.1 (12.6)	60.8 (12.4)	61.1 (12.0)
BMI	27.4 (5.8)	28.1 (5.6)	28.5 (5.1)	29.3 (5.4)
Women, %	75.7	76.0	43.0	7.0
University education, %	32.8	32.7	30.0	24.2
Physically inactive, %	20.8	17.1	16.6	21.8
Current smoking, %	21.5	17.0	18.6	20.0
Family history of diabetes, %	36.8	37.3	39.7	38.9
History of CVD or hypertension, %	34.6	39.2	44.3	46.8
Fruit (g/day)	197.1 (155.2)	173.9 (122.8)	149.9 (112.8)	112.5 (97.7)
Vegetables (g/day)	214.8 (147.0)	231.3 (144.9)	222.2 (142.4)	182.2 (132.5)
Nuts (g/day)	5.3 (7.3)	4.7 (6.5)	3.7 (5.5)	2.4 (3.6)
Fish (g/day)	42.2 (33.7)	45.9 (35.0)	47.5 (38.6)	46.4 (33.5)
Fatty fish (g/day)	18.9 (19.1)	20.9 (19.7)	21.8 (19.6)	22.5 (20.1)
Red and processed meat (g/day)	72.2 (52.0)	80.1 (49.4)	98.8 (55.6)	110.9 (59.8)
Dairy products (g/day)	356.3 (320.3)	397.1 (303.3)	457.2 (361.0)	398.8 (339.7)
Whole grain (g/day)	37.1 (22.3)	49.0 (27.8)	52.7 (30.2)	72.3 (51.4)
Sweetened beverages (g/day)	178.1 (427.9)	84.3 (204.0)	79.3 (184.4)	75.8 (198.3)
Coffee (g/day)	424.9 (329.2)	422.2 (300.0)	449.1 (332.6)	462.8 (370.3)
Alcohol (g/day)	7.1 (9.0)	6.8 (8.7)	9.1 (10.3)	11.2 (12.3)
Olive oil, %	43.9	41.5	42.7	38.7
Corn or sunflower oil, %	13.5	13.2	13.1	11.6
Rapeseed oil, %	47.2	48.7	48.3	42.4

CVD: Cardiovascular diseases

Table S7. ORs and 95% CIs of LADA in relation to antioxidant nutrient intakes

Antioxidant nutrient intake [a]	N cases/controls	Model 1[b]	Model 2[c]	Model 3[d]	Model 4[e]
**Beta-carotene (µg/day)**					
Q1 (<1642.4)	156/569	1.00	1.00	1.00	1.00
Q2 (1642.4 to <2595.6)	151/569	1.00 (0.78, 1.30)	1.14 (0.87, 1.50)	1.14 (0.87, 1.50)	1.15 (0.87, 1.51)
Q3 (2595.6 to <4072.1)	121/569	0.80 (0.61, 1.05)	0.83 (0.63, 1.10)	0.85 (0.64, 1.13)	0.86 (0.64, 1.14)
Q4 (≥4072.1)	156/569	1.06 (0.82, 1.37)	1.12 (0.86, 1.48)	1.18 (0.89, 1.57)	1.16 (0.87, 1.54)
Per 1 SD (2670)		1.02 (0.93, 1.10)	1.02 (0.93, 1.12)	1.04 (0.94, 1.15)	1.04 (0.94, 1.15)
Supplementation (ever vs never)	9/52	0.71 (0.35, 1.47)	0.78 (0.37, 1.64)	0.81 (0.39, 1.70)	0.76 (0.36, 1.60)
**Vitamin C (mg/day)**					
Q1 (<73.6)	174/569	1.00	1.00	1.00	1.00
Q2 (73.6 to <104.2)	158/569	0.93 (0.73, 1.20)	0.95 (0.73, 1.23)	0.95 (0.73, 1.23)	0.96 (0.74, 1.25)
Q3 (104.2 to <144.6)	138/569	0.81 (0.63, 1.04)	0.81 (0.62, 1.06)	0.81 (0.62, 1.06)	0.79 (0.60, 1.04)
Q4 (≥144.6)	114/569	0.67 (0.51, 0.87)	0.65 (0.49, 0.86)	0.67 (0.51, 0.90)	0.66 (0.49, 0.88)
Per 1 SD (64.2)		0.91 (0.83, 1.00)	0.90 (0.82, 1.00)	0.92 (0.83, 1.02)	0.91 (0.82, 1.01)
Supplementation (ever vs never)	65/268	0.96 (0.72, 1.28)	1.03 (0.76, 1.40)	1.02 (0.75, 1.39)	1.00 (0.73, 1.36)
**Vitamin E (mg/day)**					
Q1 (<7.2)	159/569	1.00	1.00	1.00	1.00
Q2 (7.2 to <8.7)	158/569	0.98 (0.76, 1.25)	0.98 (0.75, 1.28)	1.01 (0.77, 1.32)	1.01 (0.78, 1.33)
Q3 (8.7 to <10.6)	123/569	0.76 (0.58, 0.99)	0.75 (0.57, 1.00)	0.79 (0.60, 1.05)	0.79 (0.59, 1.05)
Q4 (≥10.6)	144/569	0.85 (0.66, 1.10)	0.81 (0.62, 1.06)	0.86 (0.65, 1.15)	0.84 (0.63, 1.13)
Per 1 SD (3.0)		0.93 (0.85, 1.02)	0.92 (0.83, 1.01)	0.94 (0.85, 1.05)	0.93 (0.84, 1.04)
Supplementation (ever vs never)	19/81	0.92 (0.55, 1.54)	0.94 (0.55, 1.60)	0.92 (0.54, 1.58)	0.91 (0.53, 1.57)
**Selenium (µg/day)**					
Q1 (<33.4)	130/569	1.00	1.00	1.00	1.00
Q2 (33.4 to <40.2)	134/569	1.01 (0.77, 1.33)	0.96 (0.72, 1.27)	0.97 (0.73, 1.20)	0.97 (0.73, 1.29)
Q3 (40.2 to <48.7)	159/569	1.15 (0.88, 1.51)	1.08 (0.81, 1.43)	1.10 (0.82, 1.46)	1.10 (0.82, 1.46)
Q4 (≥48.7)	161/569	1.14 (0.86, 1.51)	1.07 (0.79, 1.44)	1.12 (0.83, 1.51)	1.10 (0.81, 1.49)
Per 1 SD (13.5)		1.02 (0.93, 1.13)	0.99 (0.89, 1.10)	1.01 (0.90, 1.12)	1.00 (0.90, 1.11)
Supplementation (ever vs never)	18/81	0.93 (0.55, 1.57)	1.02 (0.59, 1.76)	0.90 (0.38, 2.14)	1.03 (0.59, 1.79)
**Zinc (mg/day)**					
Q1 (<8.6)	116/569	1.00	1.00	1.00	1.00
Q2 (8.6 to <9.8)	147/569	1.25 (0.95, 1.64)	1.23 (0.92, 1.63)	1.25 (0.94, 1.67)	1.24 (0.93, 1.65)
Q3 (9.8 to <11.5)	166/569	1.32 (1.00, 1.75)	1.17 (0.88, 1.57)	1.22 (0.90, 1.64)	1.19 (0.88, 1.60)
Q4 (≥11.5)	155/569	1.18 (0.86, 1.61)	0.96 (0.69, 1.33)	1.05 (0.75, 1.47)	1.00 (0.71, 1.40)
Per 1 SD (2.5)		1.08 (0.98, 1.20)	1.00 (0.90, 1.12)	1.04 (0.93, 1.16)	1.03 (0.92, 1.15)
Supplementation (ever vs never)	23/92	0.99 (0.62, 1.60)	1.05 (0.64, 1.72)	1.05 (0.64, 1.73)	1.05 (0.64, 1.73)
**Multivitamins (ever vs never)**	93/519	0.64 (0.50, 0.82)	0.71 (0.55, 0.92)	0.69 (0.54, 0.90)	0.71 (0.54, 0.92)

[a] Adjusted for energy intake except for supplementation.
[b] Adjusted for age and sex.
[c] Adjusted for Model 1 + education, physical activity, smoking, intake of alcohol, family history of diabetes, and BMI
[d] Adjusted for Model 2 + tertiles of fatty fish (except for selenium), red meat (except for selenium and zinc), sweetened beverages, and coffee intake.
[e] Adjusted for Model 3 + history of cardiovascular diseases or hypertension.

Table S8. ORs and 95% CIs of LADA with low/intermediate- or high-risk HLA genotype in relation to antioxidant nutrient intakes

**Antioxidant nutrient intake**[a]	**Low/intermediate-risk HLA**		**High-risk HLA**	
	N cases/controls	*OR (95% CI)*	*N cases/controls*	*OR (95% CI)*
**Beta-carotene (μg/day)**				
Q1 (<1642.4)	34/569	1.00	66/569	1.00
Q2 (1642.4 to <2595.6)	38/569	1.37 (0.81, 2.29)	62/569	1.14 (0.77, 1.71)
Q3 (2595.6 to <4072.1)	37/569	1.47 (0.87, 2.47)	45/569	0.81 (0.52, 1.24)
Q4 (≥4072.1)	47/569	1.80 (1.07, 3.01)	64/569	1.23 (0.81, 1.87)
Per 1 SD (2670)		1.13 (0.96, 1.32)		1.06 (0.90, 1.25)
Supplementation (ever vs never)	2/52	0.56 (0.13, 2.51)	4/52	0.70 (0.23, 2.11)
**Vitamin C (mg/day)**				
Q1 (<73.6)	41/569	1.00	71/569	1.00
Q2 (73.6 to <104.2)	45/569	1.11 (0.69, 1.80)	68/569	1.06 (0.72, 1.57)
Q3 (104.2 to <144.6)	43/569	1.11 (0.68, 1.82)	50/569	0.74 (0.48, 1.12)
Q4 (≥144.6)	27/569	0.68 (0.39, 1.18)	48/569	0.77 (0.50, 1.19)
Per 1 SD (64.2)		0.95 (0.80, 1.15)		0.94 (0.80, 1.11)
Supplementation (ever vs never)	19/268	1.11 (0.64, 1.93)	22/268	0.82 (0.49, 1.35)
**Vitamin E (mg/day)**				
Q1 (<7.2)	40/569	1.00	67/569	1.00
Q2 (7.2 to <8.7)	39/569	0.97 (0.59, 1.58)	56/569	0.88 (0.59, 1.32)
Q3 (8.7 to <10.6)	35/569	1.03 (0.62, 1.73)	56/569	0.92 (0.61, 1.39)
Q4 (≥10.6)	42/569	1.13 (0.66, 1.92)	58/569	0.99 (0.64, 1.54)
Per 1 SD (3.0)		1.07 (0.87, 1.30)		1.00 (0.85, 1.19)
Supplementation (ever vs never)	7/81	1.20 (0.51, 2.84)	8/81	0.99 (0.44, 2.27)
**Selenium (μg/day)**				
Q1 (<33.4)	36/569	1.00	50/569	1.00
Q2 (33.4 to <40.2)	34/569	0.86 (0.51, 1.44)	50/569	1.03 (0.67, 1.60)
Q3 (40.2 to <48.7)	45/569	1.16 (0.70, 1.93)	67/569	1.36 (0.89, 2.09)
Q4 (≥48.7)	41/569	1.12 (0.64, 1.94)	70/569	1.54 (0.98, 2.42)
Per 1 SD (13.5)		1.08 (0.90, 1.30)		1.12 (0.96, 1.31)
Supplementation (ever vs never)	5/81	0.93 (0.34, 2.51)	7/81	0.90 (0.38, 2.14)
**Zinc (mg/day)**				
Q1 (<8.6)	25/569	1.00	43/569	1.00
Q2 (8.6 to <9.8)	43/569	1.74 (1.01, 2.98)	58/569	1.46 (0.93, 2.27)
Q3 (9.8 to <11.5)	42/569	1.65 (0.94, 2.92)	72/569	1.71 (1.09, 2.68)
Q4 (≥11.5)	46/569	1.78 (0.94, 3.36)	64/569	1.38 (0.82, 2.32)
Per 1 SD (2.5)		1.14 (0.93, 1.40)		1.17 (0.99, 1.39)
Supplementation (ever vs never)	3/92	0.51 (0.15, 1.76)	5/92	0.62 (0.24, 1.65)
**Multivitamins (ever vs never)**	23/519	0.64 (0.39, 1.04)	39/519	0.70 (0.48, 1.04)

[a]Adjusted for energy intake except for supplementation.

ORs adjusted for age, sex, education, physical activity, smoking, intake of alcohol, family history of diabetes, BMI, tertiles of fatty fish (except for selenium), red meat (except for selenium and zinc), sweetened beverages, and coffee intake, and history of cardiovascular diseases or hypertension.

Table S9. ORs and 95% CIs of type 2 diabetes in relation to antioxidant nutrient intakes

Antioxidant nutrient intake [a]	N cases/controls	Model 1[b]	Model 2[c]	Model 3[d]	Model 4[e]
**Beta -carotene (µg/day)**					
Q1 (<1642.4)	556/569	1.00	1.00	1.00	1.00
Q2 (1642.4 to <2595.6)	422/569	0.78 (0.65, 0.93)	0.94 (0.76, 1.16)	0.93 (0.75, 1.16)	0.91 (0.73, 1.13)
Q3 (2595.6 to <4072.1)	428/569	0.80 (0.67, 0.95)	0.91 (0.73, 1.12)	0.92 (0.74, 1.14)	0.89 (0.71, 1.11)
Q4 (≥4072.1)	583/569	1.10 (0.92, 1.30)	1.19 (0.96, 1.46)	1.21 (0.98, 1.51)	1.15 (0.92, 1.43)
Per 1 SD (2670)		1.07 (1.01, 1.14)	1.06 (0.99, 1.14)	1.09 (1.01, 1.18)	1.09 (1.01, 1.17)
Supplementation (ever vs never)	36/52	0.90 (0.58, 1.40)	1.16 (0.68, 1.97)	1.18 (0.68, 2.03)	1.11 (0.63, 1.94)
**Vitamin C (mg/day)**					
Q1 (<73.6)	537/569	1.00	1.00	1.00	1.00
Q2 (73.6 to <104.2)	487/569	0.87 (0.73, 1.04)	0.96 (0.78, 1.18)	0.94 (0.76, 1.16)	0.93 (0.75, 1.15)
Q3 (104.2 to <144.6)	439/569	0.81 (0.68, 0.97)	0.87 (0.70, 1.08)	0.86 (0.69, 1.07)	0.82 (0.65, 1.02)
Q4 (≥144.6)	526/569	0.98 (0.82, 1.16)	1.05 (0.85, 1.30)	1.05 (0.85, 1.31)	0.99 (0.80, 1.24)
Per 1 SD (64.2)		1.04 (0.98, 1.10)	1.05 (0.98, 1.12)	1.07 (1.00, 1.15)	1.05 (0.98, 1.13)
Supplementation (ever vs never)	180/268	0.79 (0.65, 0.98)	0.87 (0.68, 1.12)	0.85 (0.66, 1.10)	0.85 (0.65, 1.10)
**Vitamin E (mg/day)**					
Q1 (<7.2)	467/569	1.00	1.00	1.00	1.00
Q2 (7.2 to <8.7)	393/569	0.82 (0.68, 0.98)	0.85 (0.68, 1.06)	0.89 (0.71, 1.11)	0.87 (0.69, 1.09)
Q3 (8.7 to <10.6)	497/569	0.95 (0.79, 1.13)	0.95 (0.77, 1.18)	0.98 (0.78, 1.22)	0.96 (0.77, 1.20)
Q4 (≥10.6)	632/569	1.12 (0.94, 1.34)	1.04 (0.84, 1.29)	1.08 (0.86, 1.36)	1.03 (0.82, 1.30)
Per 1 SD (3.0)		1.06 (1.00, 1.12)	1.03 (0.96, 1.10)	1.05 (0.97, 1.13)	1.03 (0.95, 1.12)
Supplementation (ever vs never)	67/81	1.03 (0.73, 1.44)	1.08 (0.71, 1.64)	1.06 (0.69, 1.62)	1.05 (0.67, 1.63)
**Selenium (µg/day)**					
Q1 (<33.4)	375/569	1.00	1.00	1.00	1.00
Q2 (33.4 to <40.2)	422/569	1.09 (0.90, 1.31)	1.03 (0.82, 1.29)	1.05 (0.84, 1.32)	1.03 (0.81, 1.30)
Q3 (40.2 to <48.7)	512/569	1.19 (0.98, 1.43)	1.08 (0.86, 1.36)	1.11 (0.89, 1.40)	1.11 (0.88, 1.41)
Q4 (≥48.7)	680/569	1.46 (1.21, 1.77)	1.29 (1.02, 1.62)	1.33 (1.05, 1.68)	1.29 (1.01, 1.64)
Per 1 SD (13.5)		1.14 (1.07, 1.22)	1.08 (1.00, 1.17)	1.10 (1.01, 1.19)	1.08 (1.00, 1.18)
Supplementation (ever vs never)	48/81	0.69 (0.48, 1.01)	0.87 (0.56, 1.36)	1.03 (0.59, 1.78)	0.91 (0.57, 1.45)
**Zinc (mg/day)**					
Q1 (<8.6)	325/569	1.00	1.00	1.00	1.00
Q2 (8.6 to <9.8)	420/569	1.26 (1.04, 1.52)	1.26 (1.00, 1.58)	1.30 (1.03, 1.64)	1.28 (1.01, 1.63)
Q3 (9.8 to <11.5)	515/569	1.46 (1.21, 1.77)	1.30 (1.03, 1.64)	1.35 (1.06, 1.70)	1.27 (1.00, 1.61)
Q4 (≥11.5)	729/569	1.84 (1.49, 2.26)	1.35 (1.05, 1.73)	1.42 (1.10, 1.84)	1.34 (1.03, 1.74)
Per 1 SD (2.5)		1.28 (1.20, 1.37)	1.14 (1.06, 1.23)	1.16 (1.07, 1.26)	1.15 (1.06, 1.25)
Supplementation (ever vs never)	61/92	0.85 (0.60, 1.19)	1.00 (0.66, 1.50)	0.97 (0.64, 1.46)	1.01 (0.67, 1.54)
**Multivitamins (ever vs never)**	284/519	0.63 (0.54, 0.75)	0.76 (0.63, 0.93)	0.75 (0.62, 0.92)	0.76 (0.61, 0.93)

[a] Adjusted for energy intake except for supplementation.

[b] Adjusted for age and sex.

[c] Adjusted for Model 1 + education, physical activity, smoking, intake of alcohol, family history of diabetes, and BMI.

[d] Adjusted for Model 2 + tertiles of fatty fish (except for selenium), red meat (except for selenium and zinc), sweetened beverages, and coffee intake.

[e] Adjusted for Model 3 + history of cardiovascular diseases or hypertension.

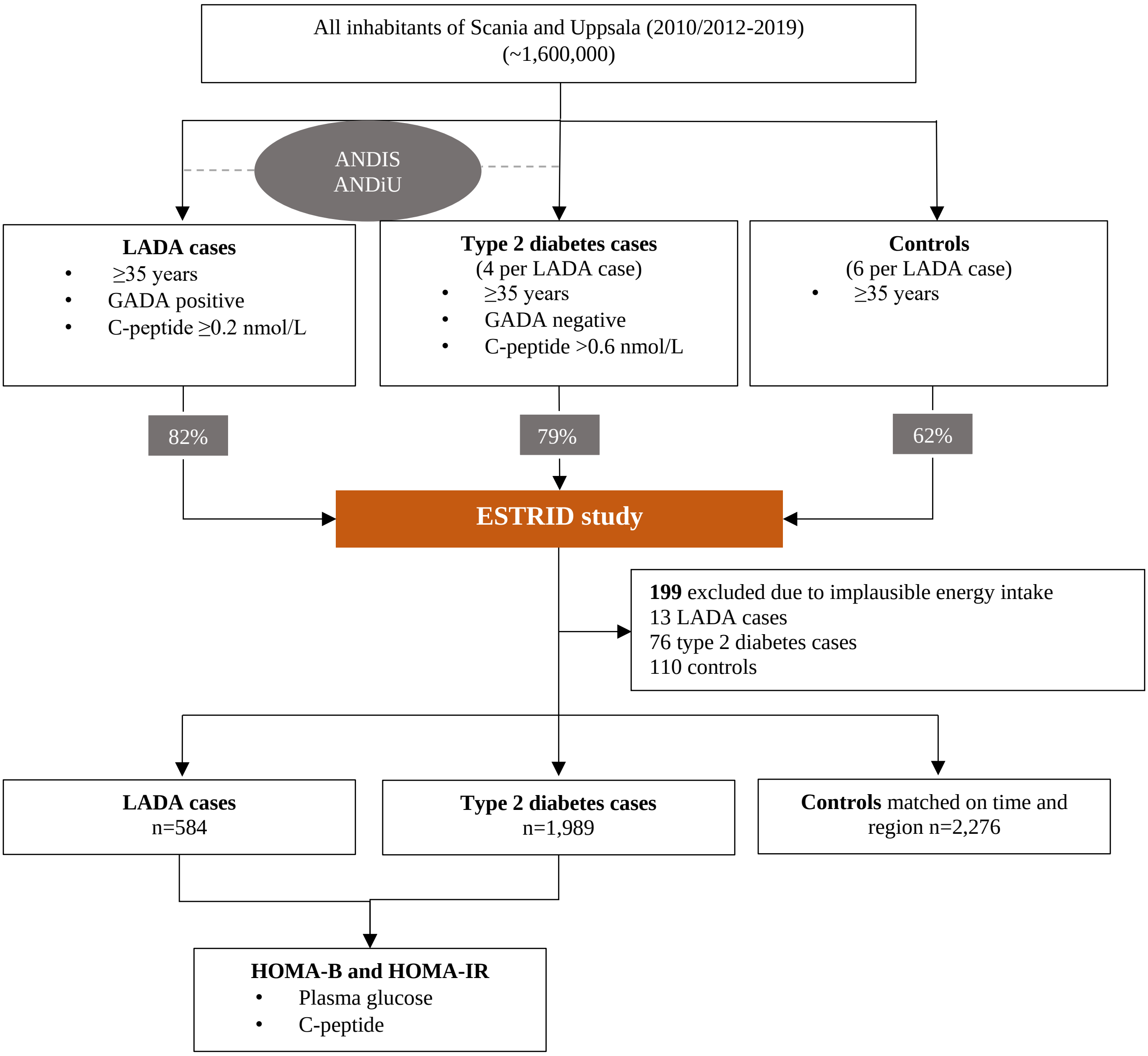

Figure S1. ESTRID case-control design and flow-diagram of study participants

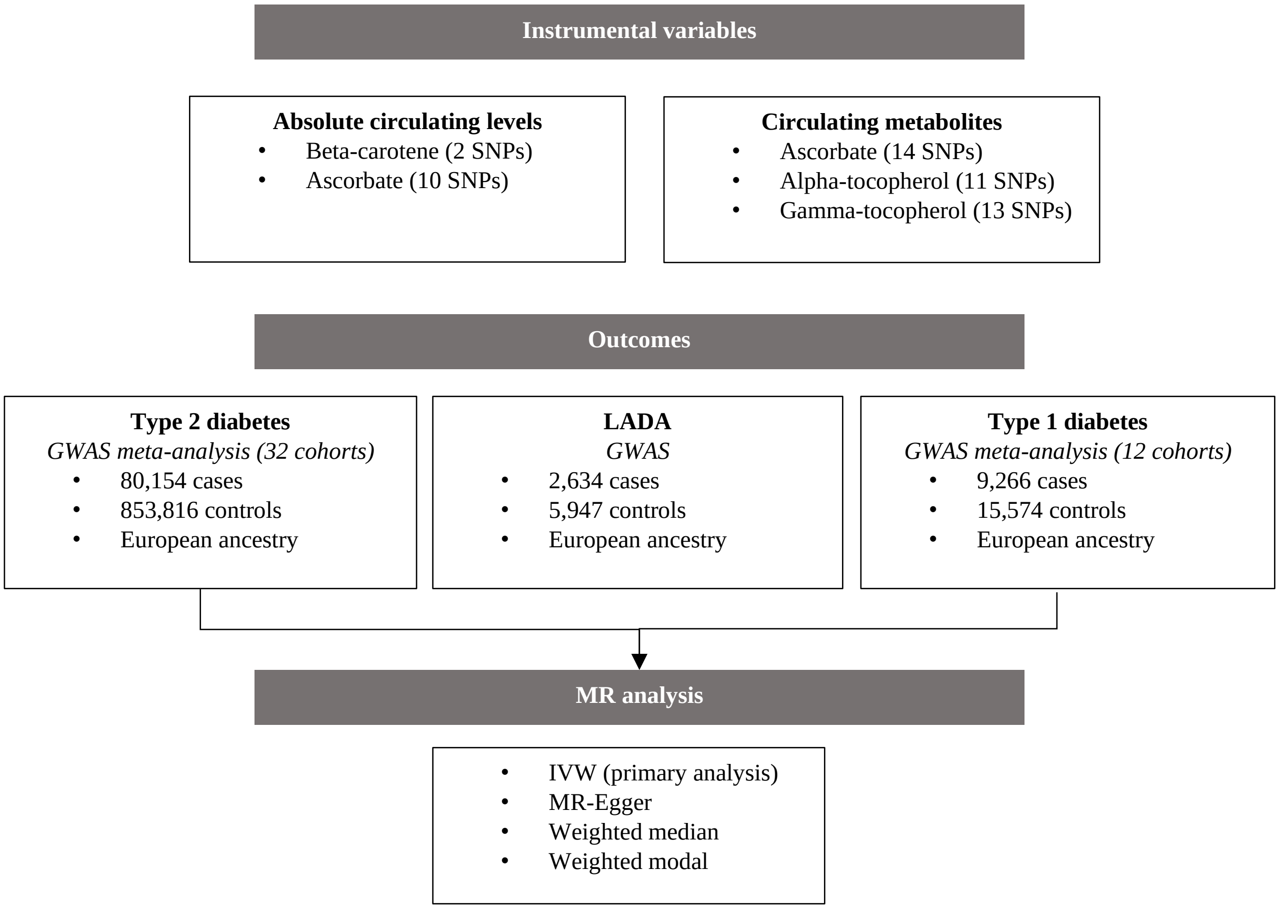

Figure S2. Flow-diagram of two-sample Mendelian randomization analyses

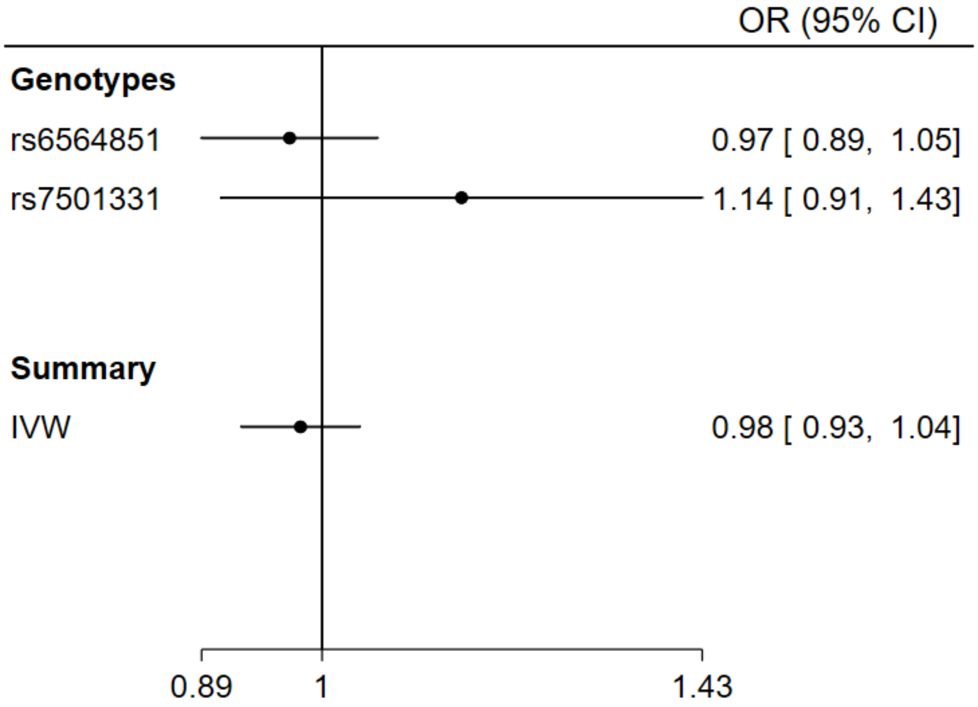

Figure S3. Two-sample Mendelian randomization analysis on the association between genetically predicted circulating beta-carotene and type 2 diabetes.

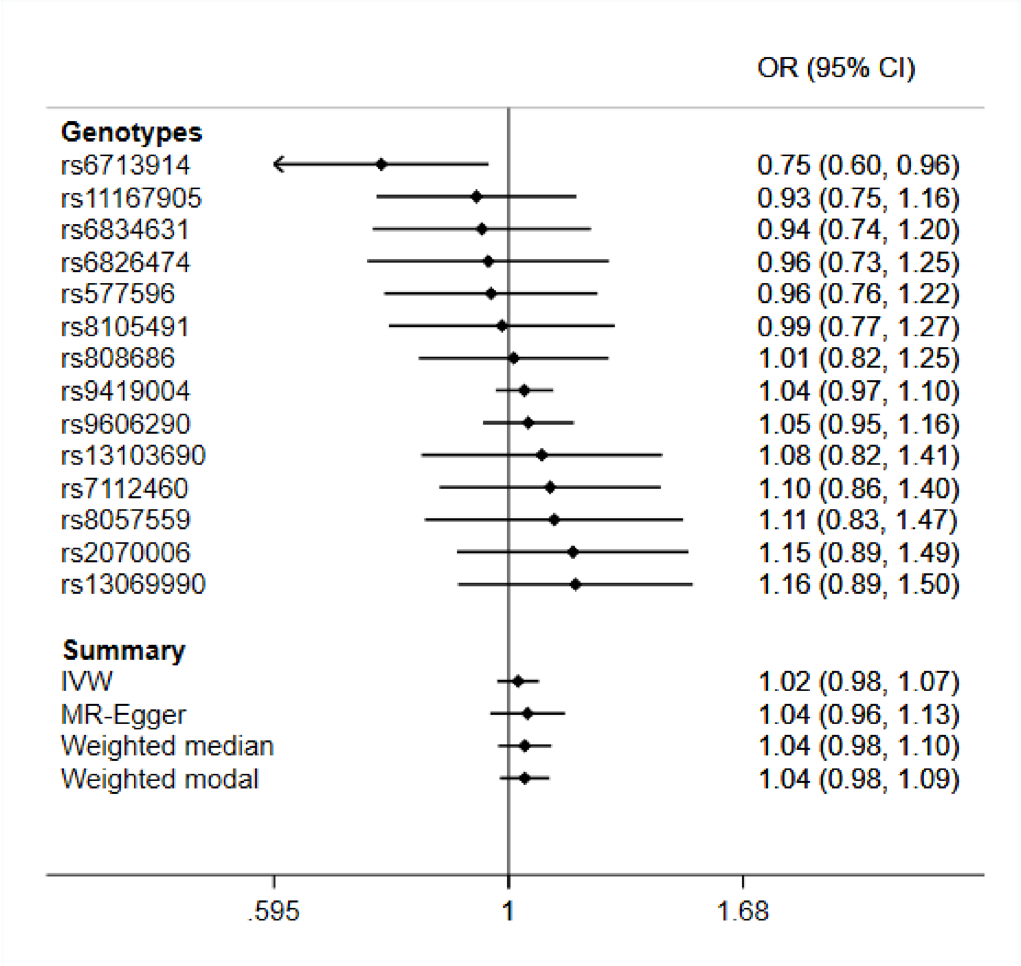

Figure S4. Two-sample Mendelian randomization analysis on the association between genetically predicted circulating vitamin C (ascorbate) metabolites and type 2 diabetes. P-value for horizontal pleiotropy = 0.507.

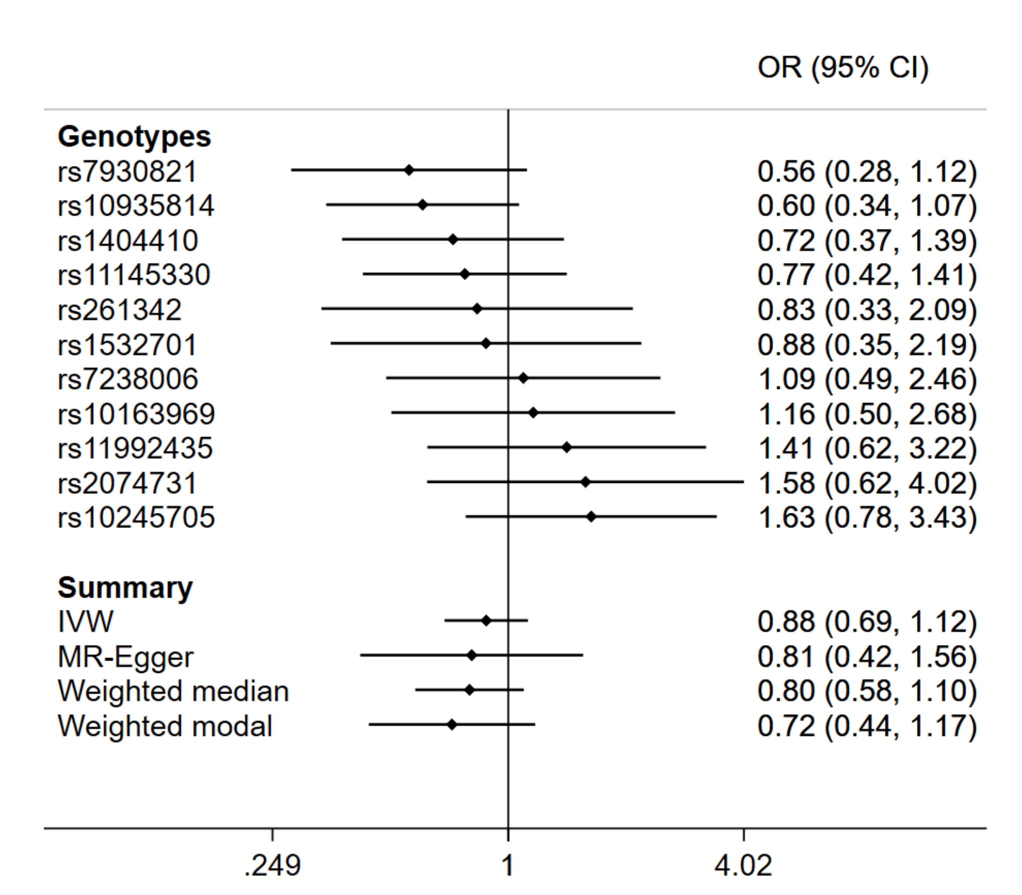

Figure S5. Two-sample Mendelian randomization analysis on the association between genetically predicted circulating vitamin E (alpha-tocopherol) metabolites and type 2 diabetes. P-value for horizontal pleiotropy = 0.745.

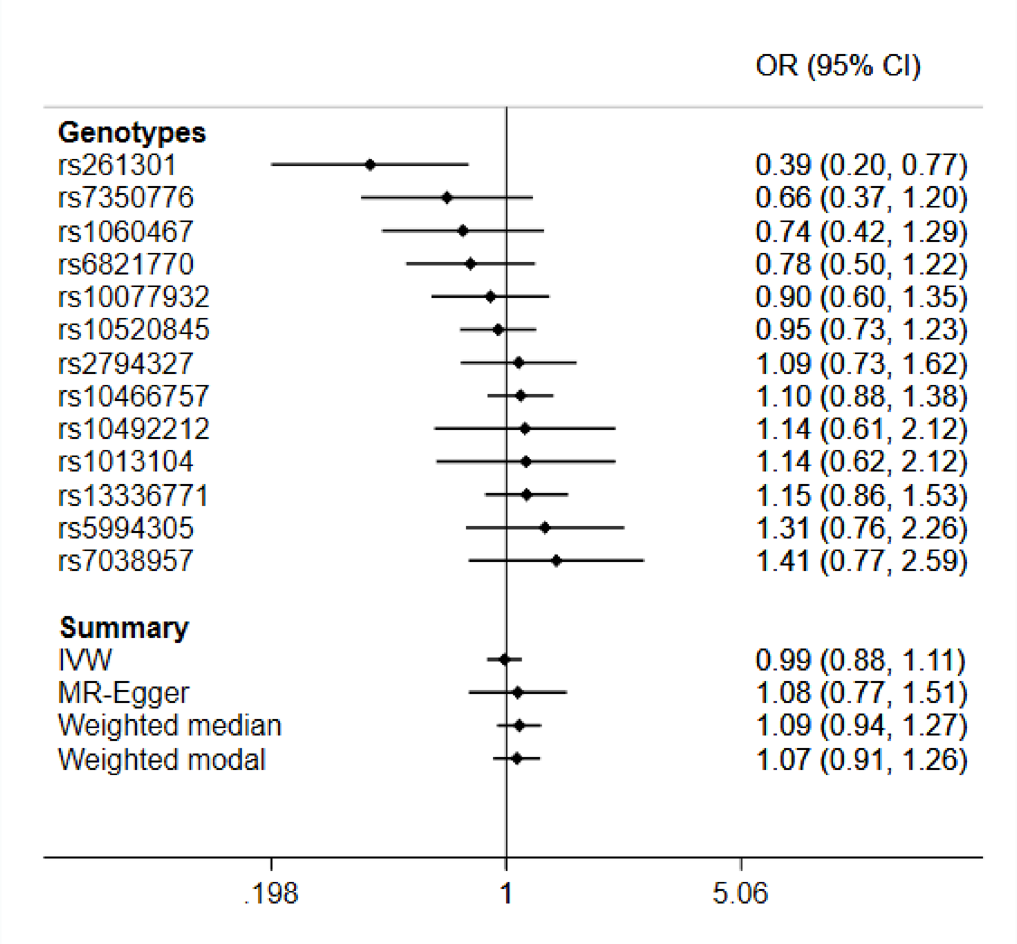

Figure S6. Two-sample Mendelian randomization analysis on the association between genetically predicted circulating vitamin E (gamma-tocopherol) metabolites and type 2 diabetes. P-value for horizontal pleiotropy = 0.496.

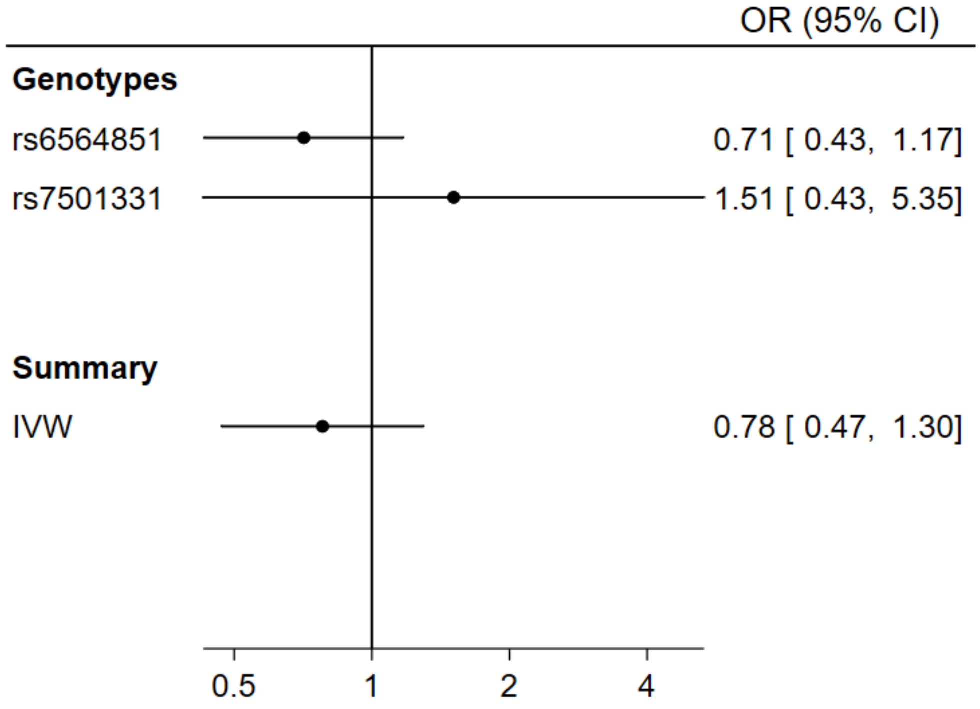

Figure S7. Two-sample Mendelian randomization analysis on the association between genetically predicted circulating beta-carotene and LADA.

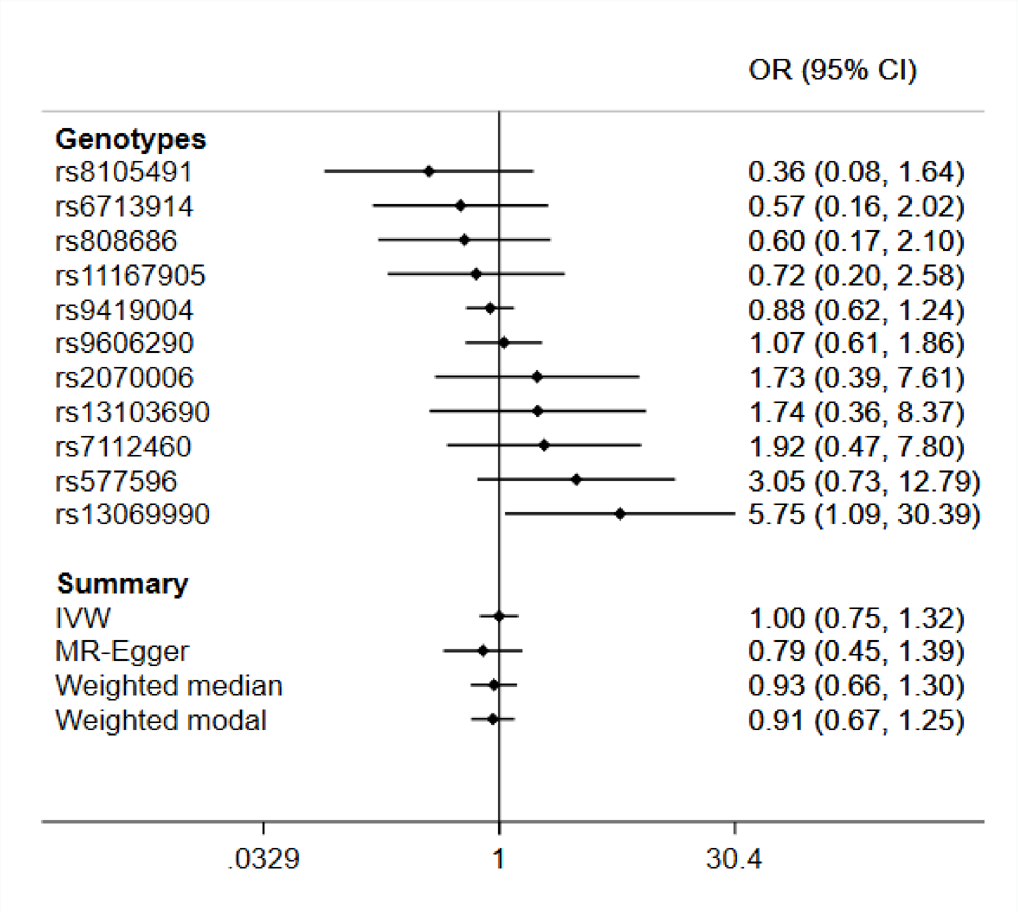

Figure S8. Two-sample Mendelian randomization analysis on the association between genetically predicted circulating vitamin C (ascorbate) metabolites and LADA. P-value for horizontal pleiotropy = 0.254. Three SNPs (rs6834631, rs8057559, rs6826474) were unavailable in the GWAS of LADA and were, thus, excluded from this MR analysis.

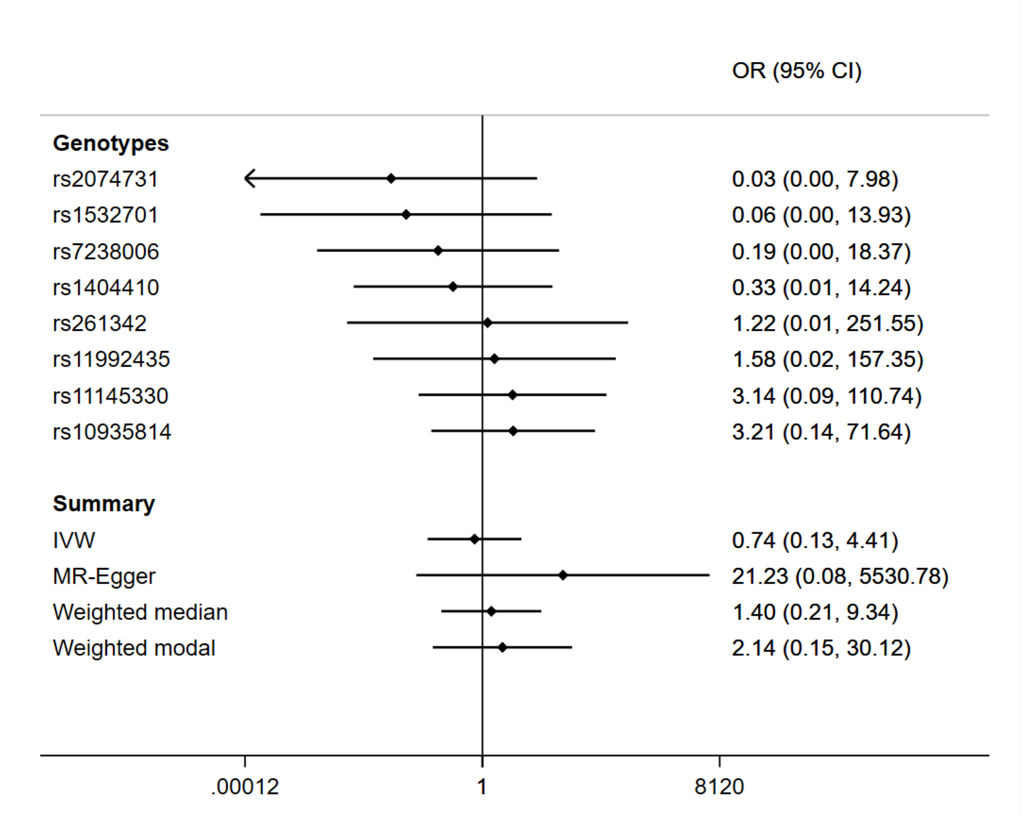

Figure S9. Two-sample Mendelian randomization analysis on the association between genetically predicted circulating vitamin E (alpha-tocopherol) metabolites and LADA. P-value for horizontal pleiotropy = 0.118. Three SNPs (rs10163969, rs10245705, rs7930821) were unavailable in the GWAS of LADA and were, thus, excluded from this MR analysis.

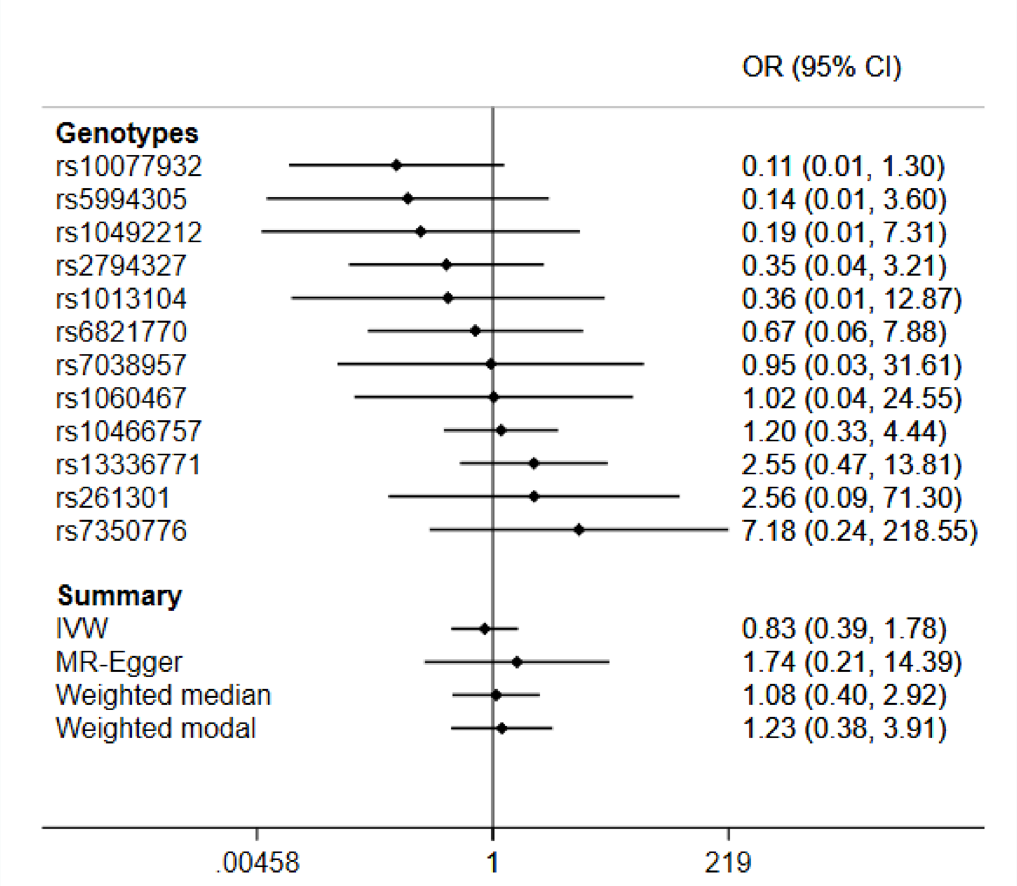

Figure S10. Two-sample Mendelian randomization analysis on the association between genetically predicted circulating vitamin E (gamma-tocopherol) metabolites and LADA. P-value for horizontal pleiotropy = 0.404. One SNP (rs10520845) was unavailable in the GWAS of LADA and was, thus, excluded from this MR analysis.

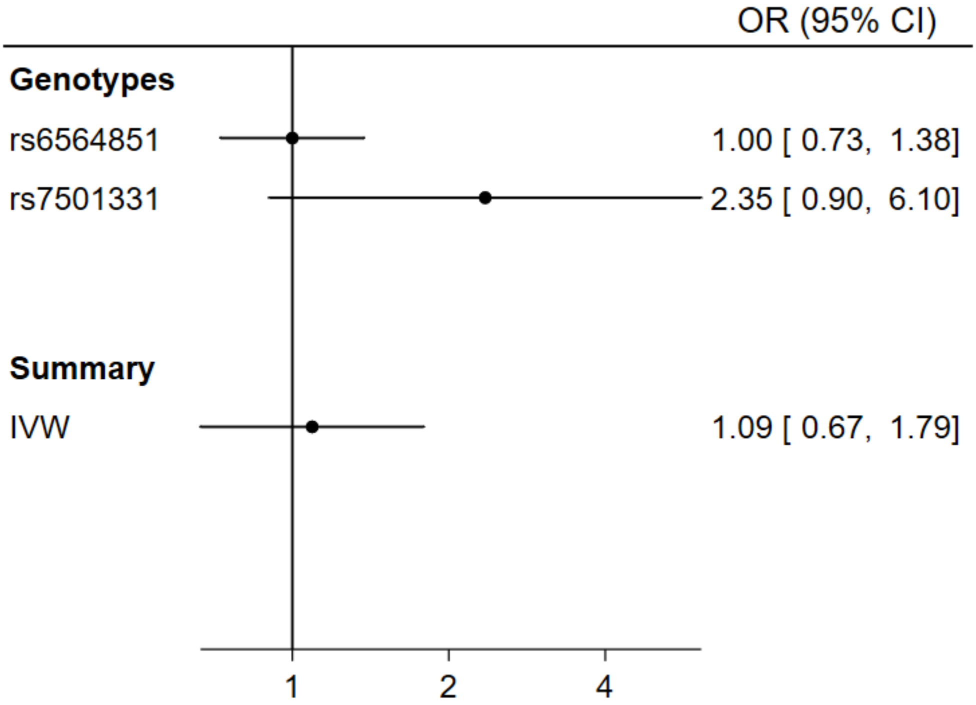

Figure S11. Two-sample Mendelian randomization analysis on the association between genetically predicted circulating beta-carotene and type 1 diabetes.

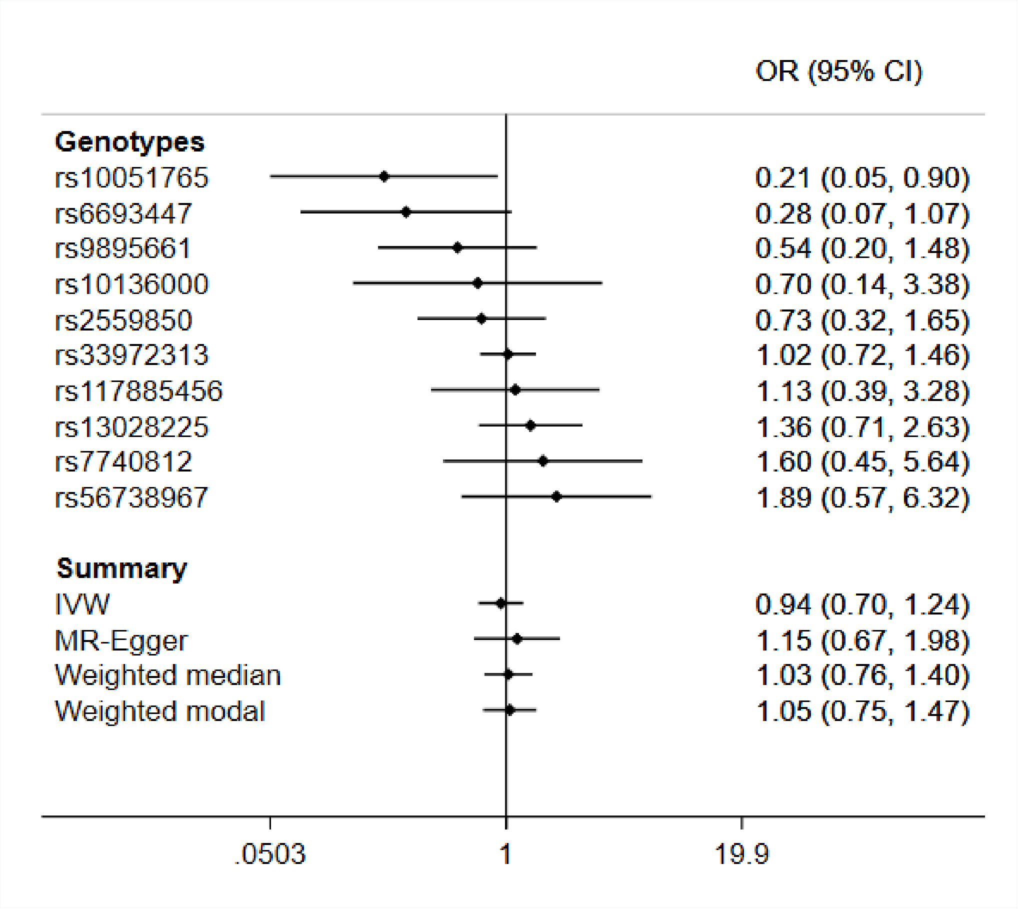

Figure S12. Two-sample Mendelian randomization analysis on the association between genetically predicted circulating vitamin C (ascorbate) and type 1 diabetes. P-value for horizontal pleiotropy = 0.259. One SNP (rs174547) with pleiotropic effect, i.e., associated also with glycerophospholipid and sphingolipid concentrations was excluded from the MR analysis.

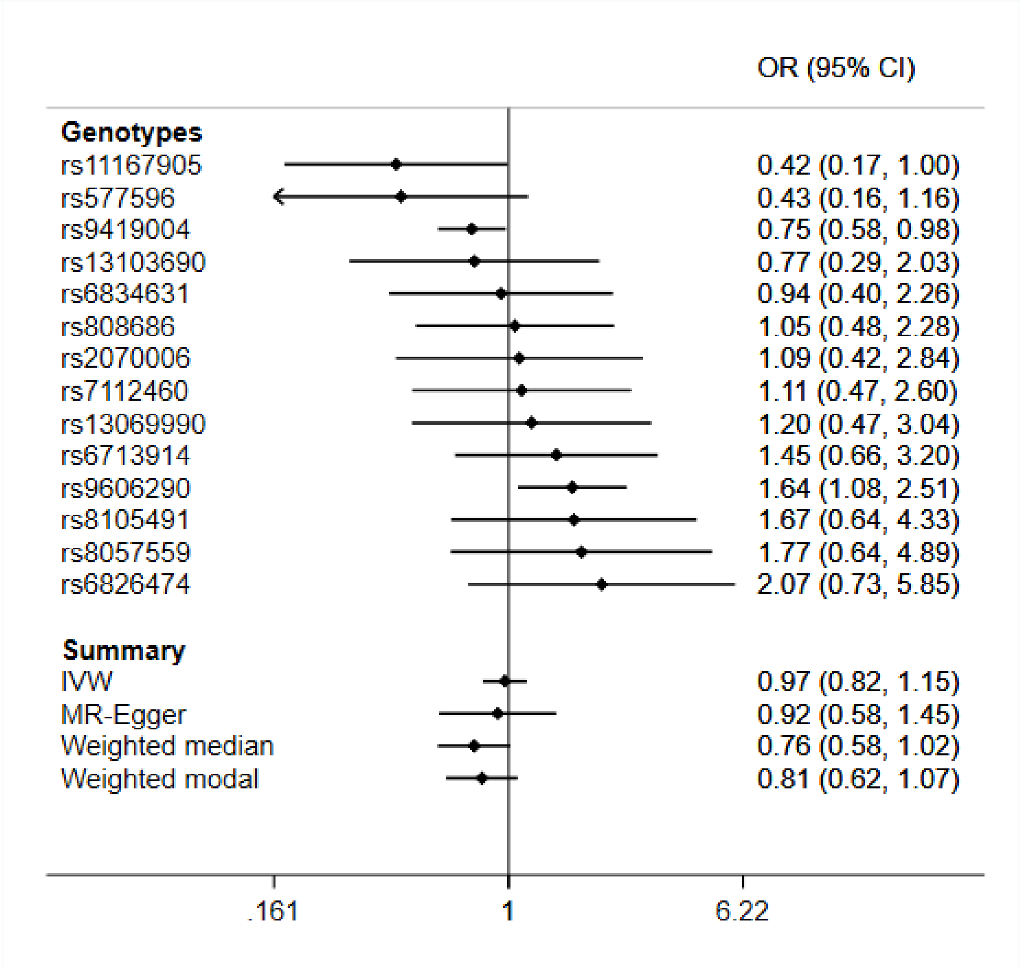

Figure S13. Two-sample Mendelian randomization analysis on the association between genetically predicted circulating vitamin C (ascorbate) metabolites and type 1 diabetes. P-value for horizontal pleiotropy = 0.747.

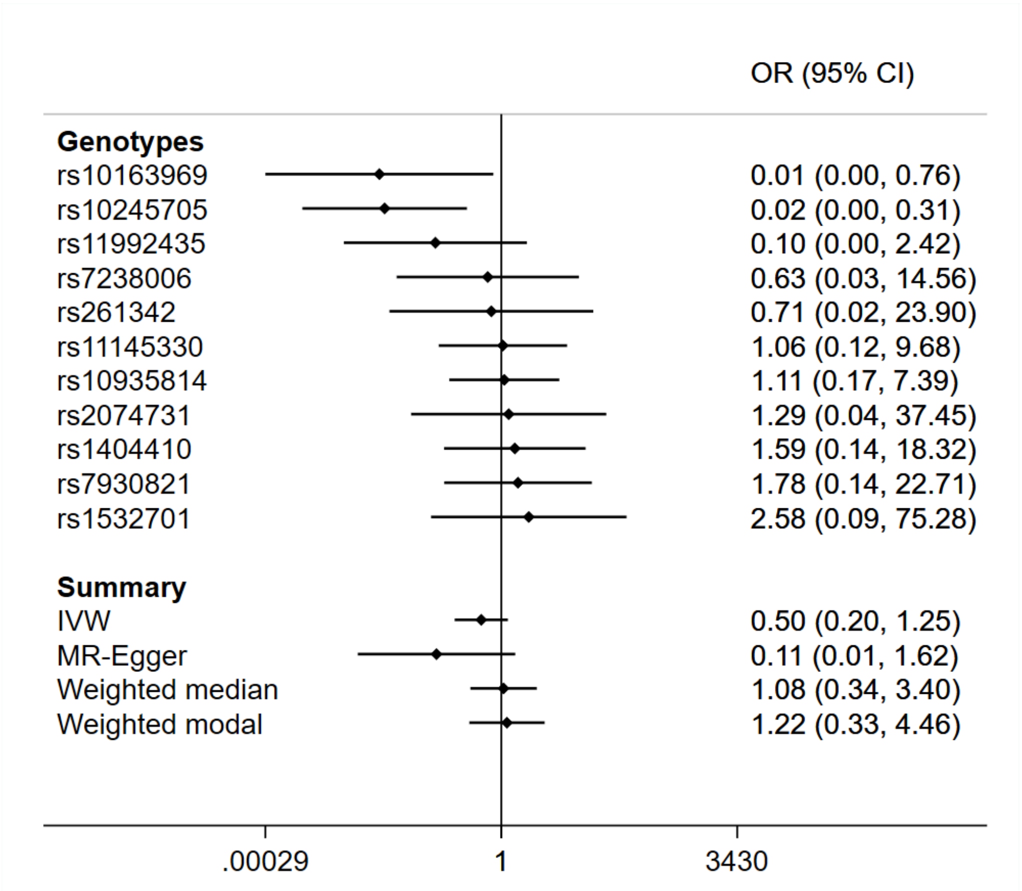

Figure S14. Two-sample Mendelian randomization analysis on the association between genetically predicted circulating vitamin E (alpha-tocopherol) metabolites and type 1 diabetes. P-value for horizontal pleiotropy = 0.156.

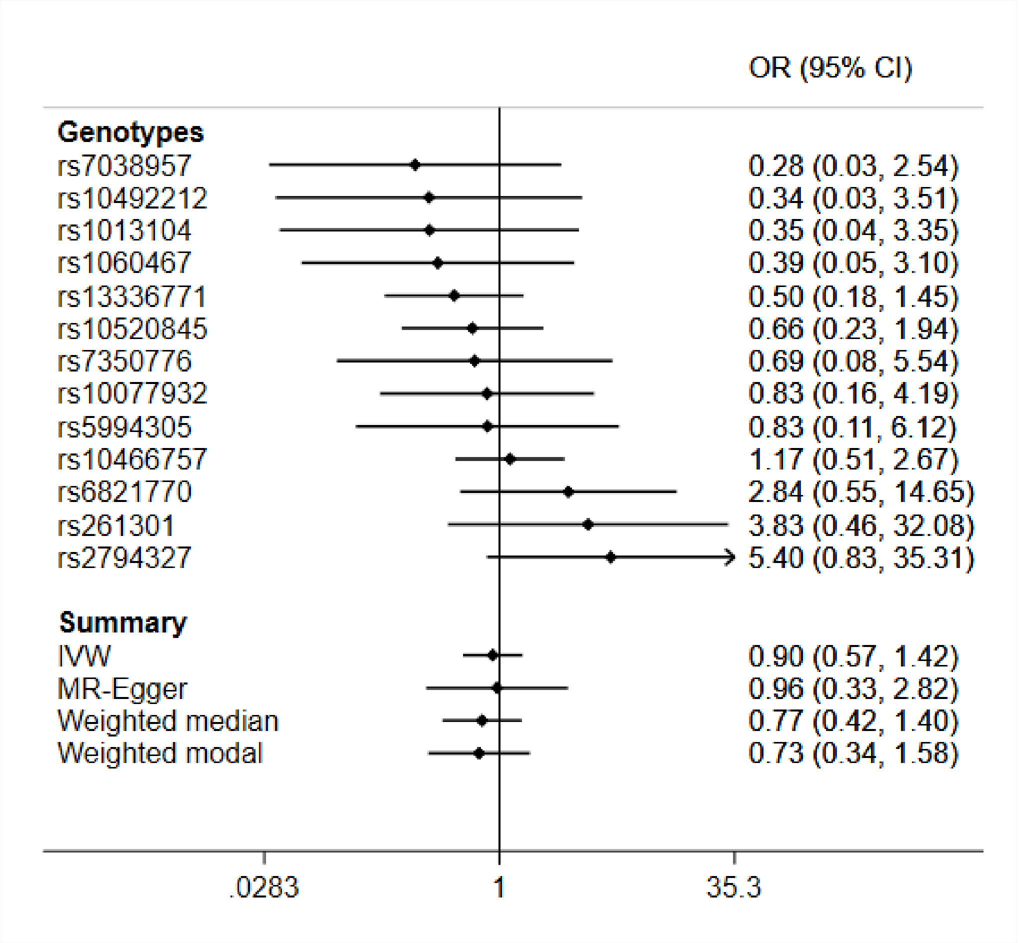

Figure S15. Two-sample Mendelian randomization analysis on the association between genetically predicted circulating vitamin E (gamma-tocopherol) metabolites and type 1 diabetes. P-value for horizontal pleiotropy = 0.880.

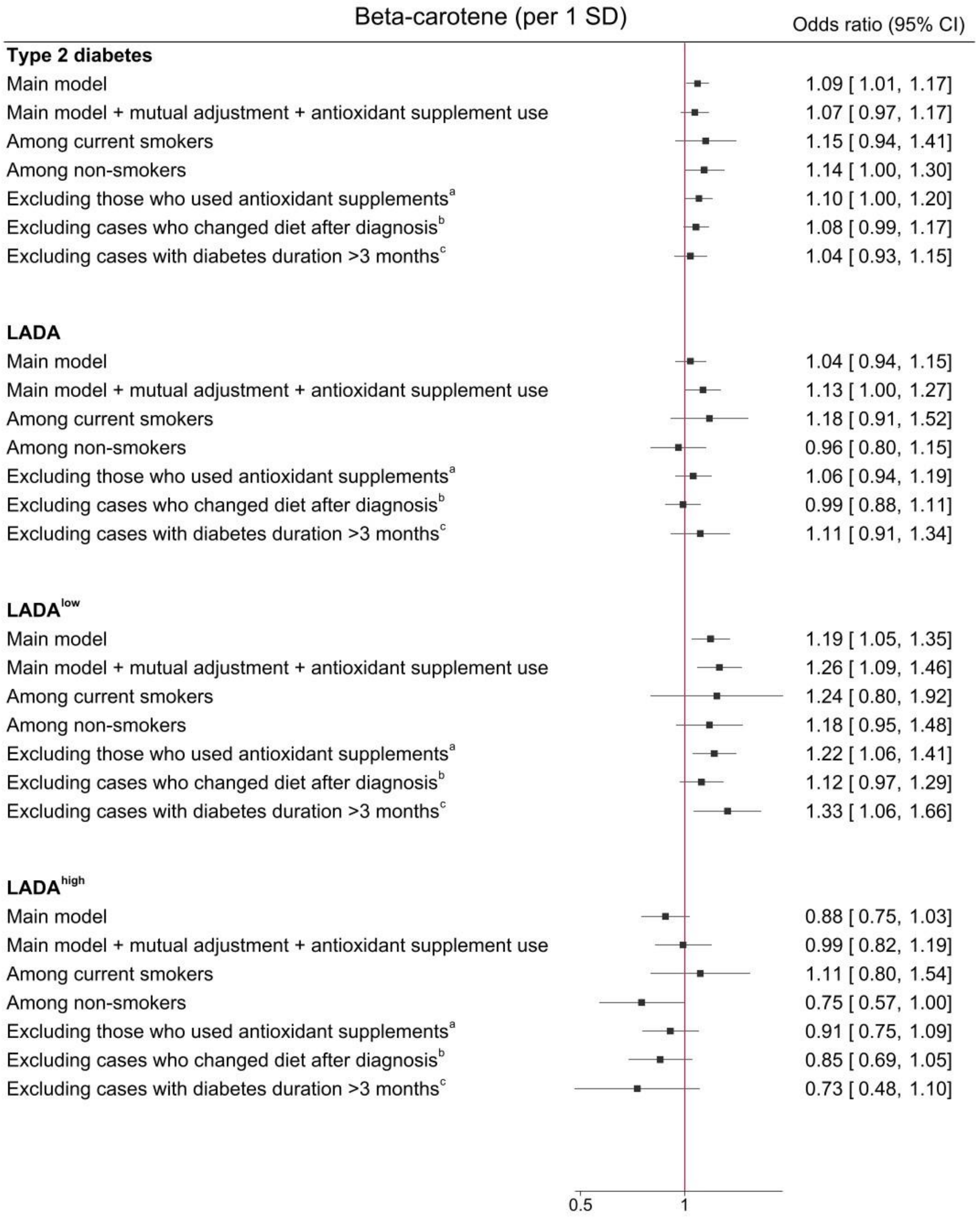

Figure S16. ORs and 95% CIs of type 2 diabetes, LADA, LADA^{low}, and LADA^{high} in relation to 1 SD higher beta-carotene intakes, in different sensitivity analyses. Main model: adjusted for age, sex, education, physical activity, smoking, intake of alcohol, family history of diabetes, BMI, energy intake, tertiles of fatty fish (except for selenium), red meat (except for selenium and zinc), sweetened beverages, and coffee intake, and history of cardiovascular diseases or hypertension. [a] n=1,240 excluded individuals. [b] n=758 excluded individuals. [c] n=1,826 excluded individuals.

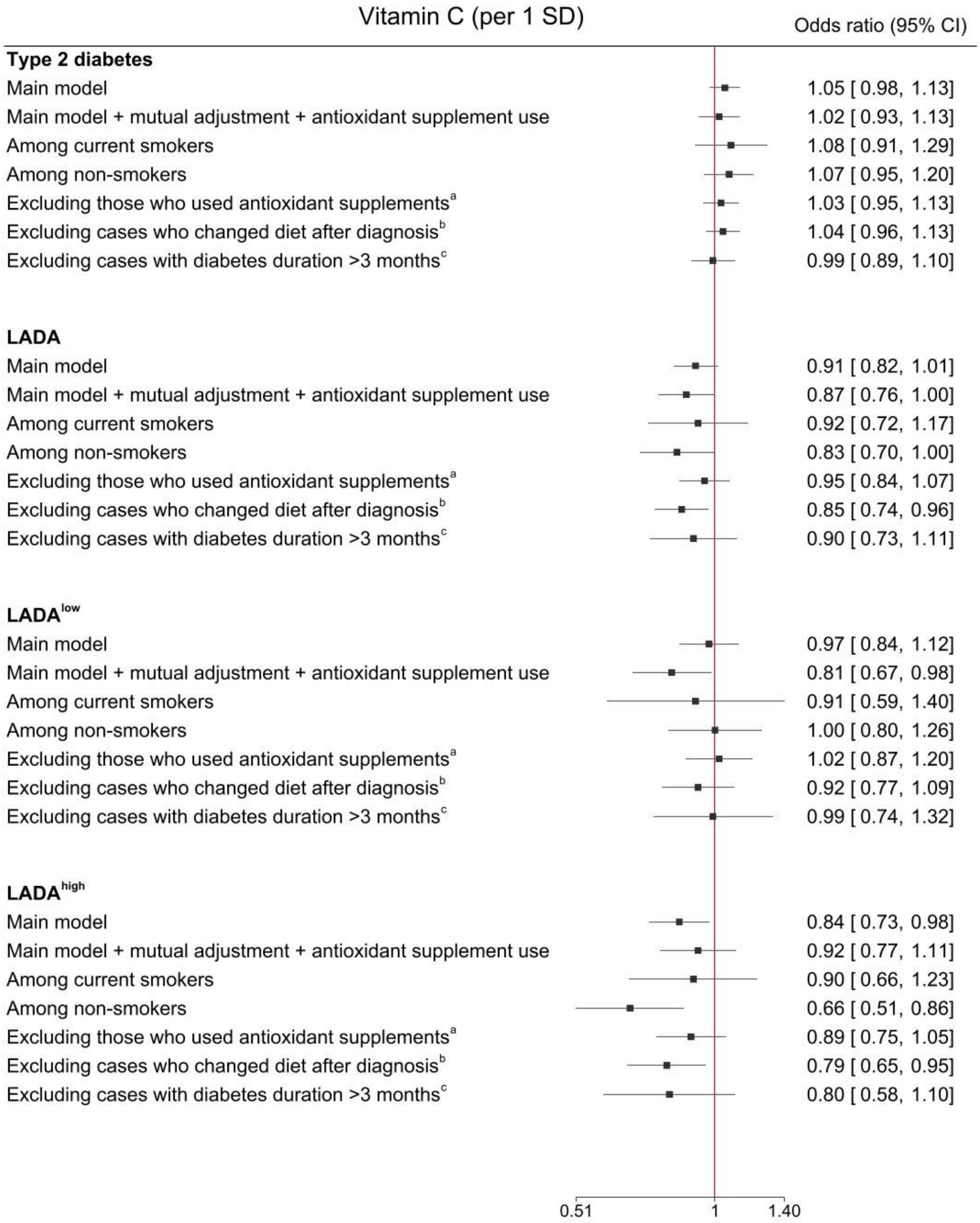

Figure S17. ORs and 95% CIs of type 2 diabetes, LADA, $LADA^{low}$, and $LADA^{high}$ in relation to 1 SD higher vitamin C intakes, in different sensitivity analyses. Main model: adjusted for age, sex, education, physical activity, smoking, intake of alcohol, family history of diabetes, BMI, energy intake, tertiles of fatty fish (except for selenium), red meat (except for selenium and zinc), sweetened beverages, and coffee intake, and history of cardiovascular diseases or hypertension. [a] n=1,240 excluded individuals. [b] n=758 excluded individuals. [c] n=1,826 excluded individuals.

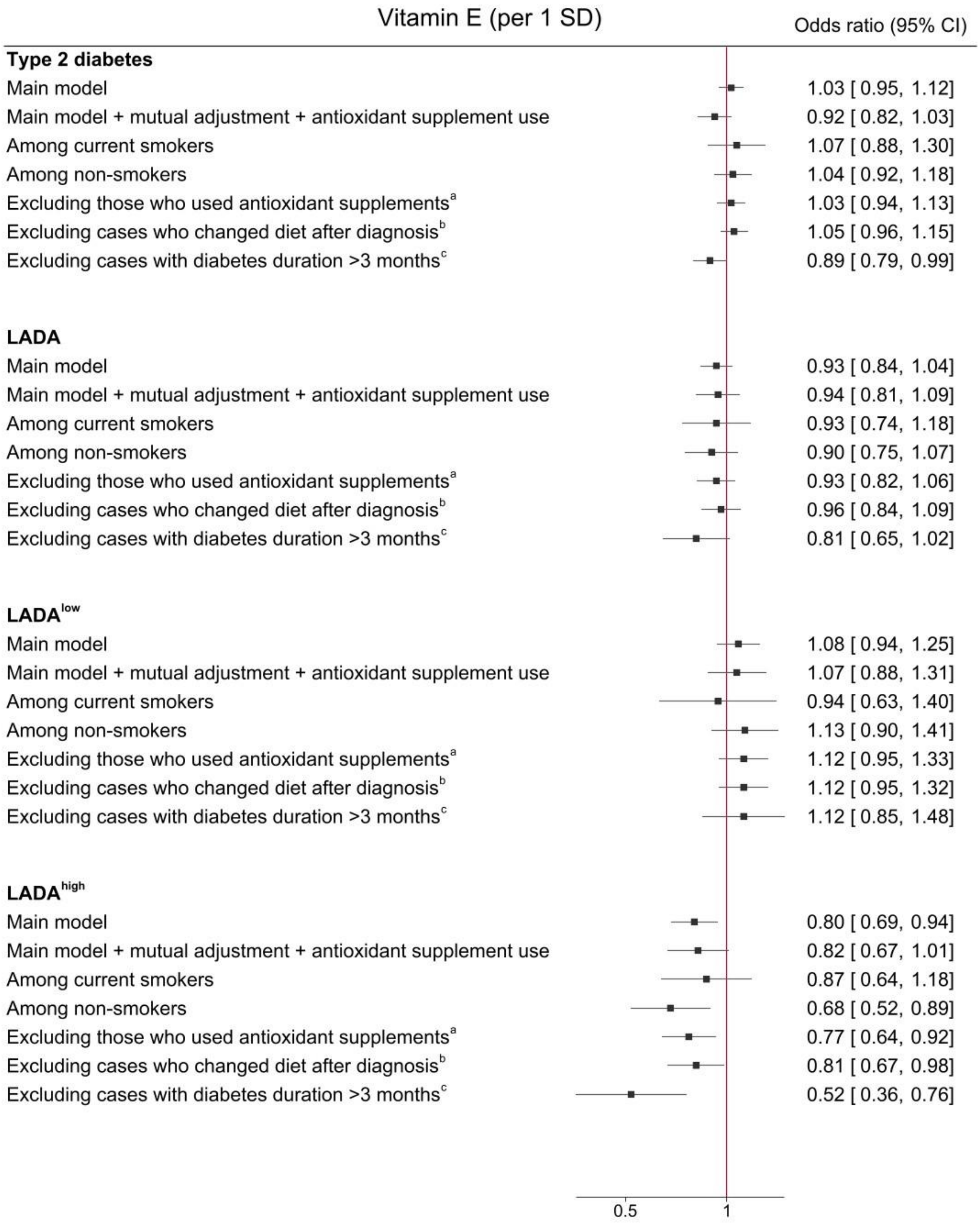

Figure S18. ORs and 95% CIs of type 2 diabetes, LADA, $LADA^{low}$, and $LADA^{high}$ in relation to 1 SD higher vitamin E intakes, in different sensitivity analyses. Main model: adjusted for age, sex, education, physical activity, smoking, intake of alcohol, family history of diabetes, BMI, energy intake, tertiles of fatty fish (except for selenium), red meat (except for selenium and zinc), sweetened beverages, and coffee intake, and history of cardiovascular diseases or hypertension. [a] n=1,240 excluded individuals. [b] n=758 excluded individuals. [c] n=1,826 excluded individuals.

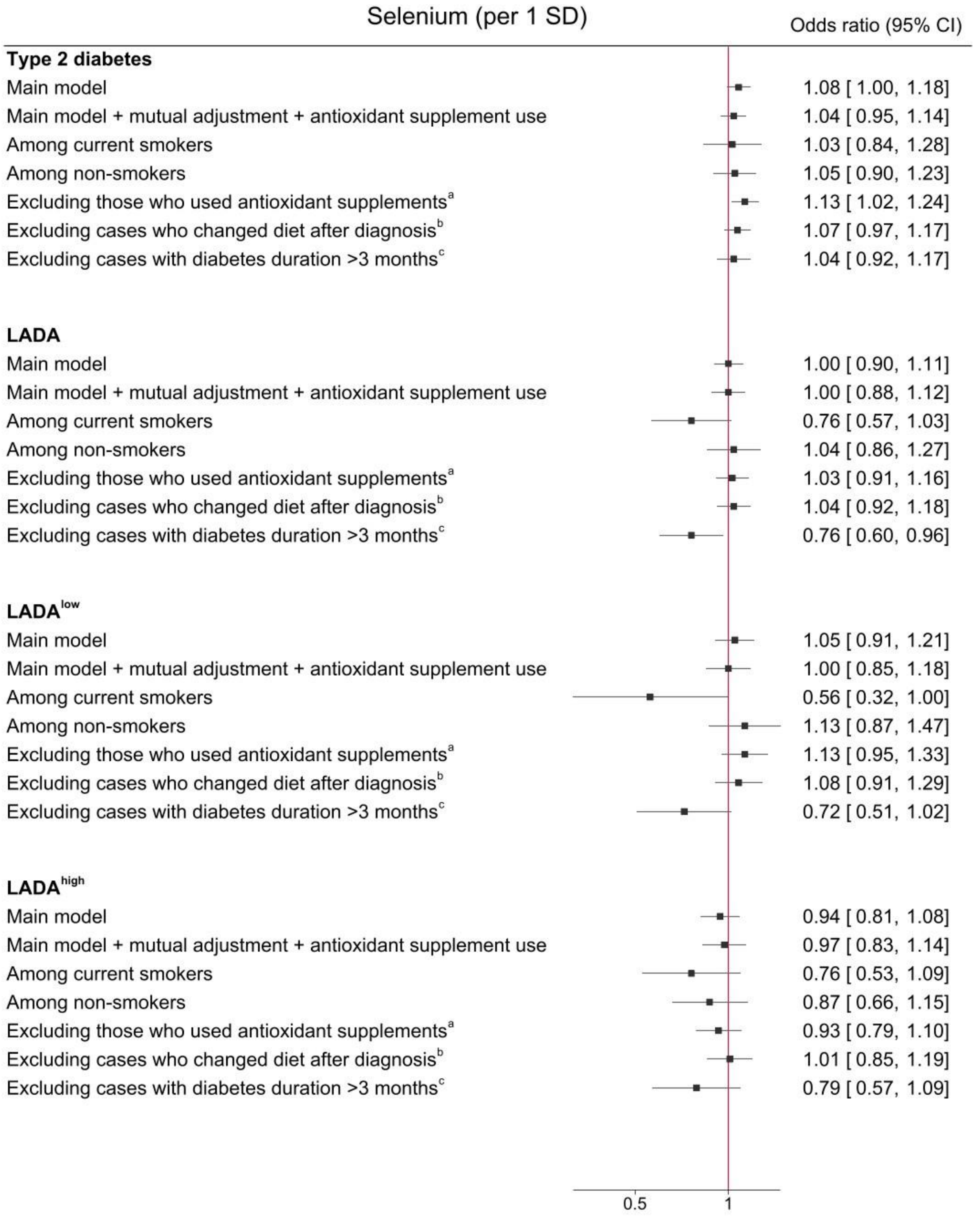

Figure S19. ORs and 95% CIs of type 2 diabetes, LADA, LADA^{low}, and LADA^{high} in relation to 1 SD higher selenium intakes, in different sensitivity analyses. Main model: adjusted for age, sex, education, physical activity, smoking, intake of alcohol, family history of diabetes, BMI, energy intake, tertiles of fatty fish (except for selenium), red meat (except for selenium and zinc), sweetened beverages, and coffee intake, and history of cardiovascular diseases or hypertension. [a] n=1,240 excluded individuals. [b] n=758 excluded individuals. [c] n=1,826 excluded individuals.

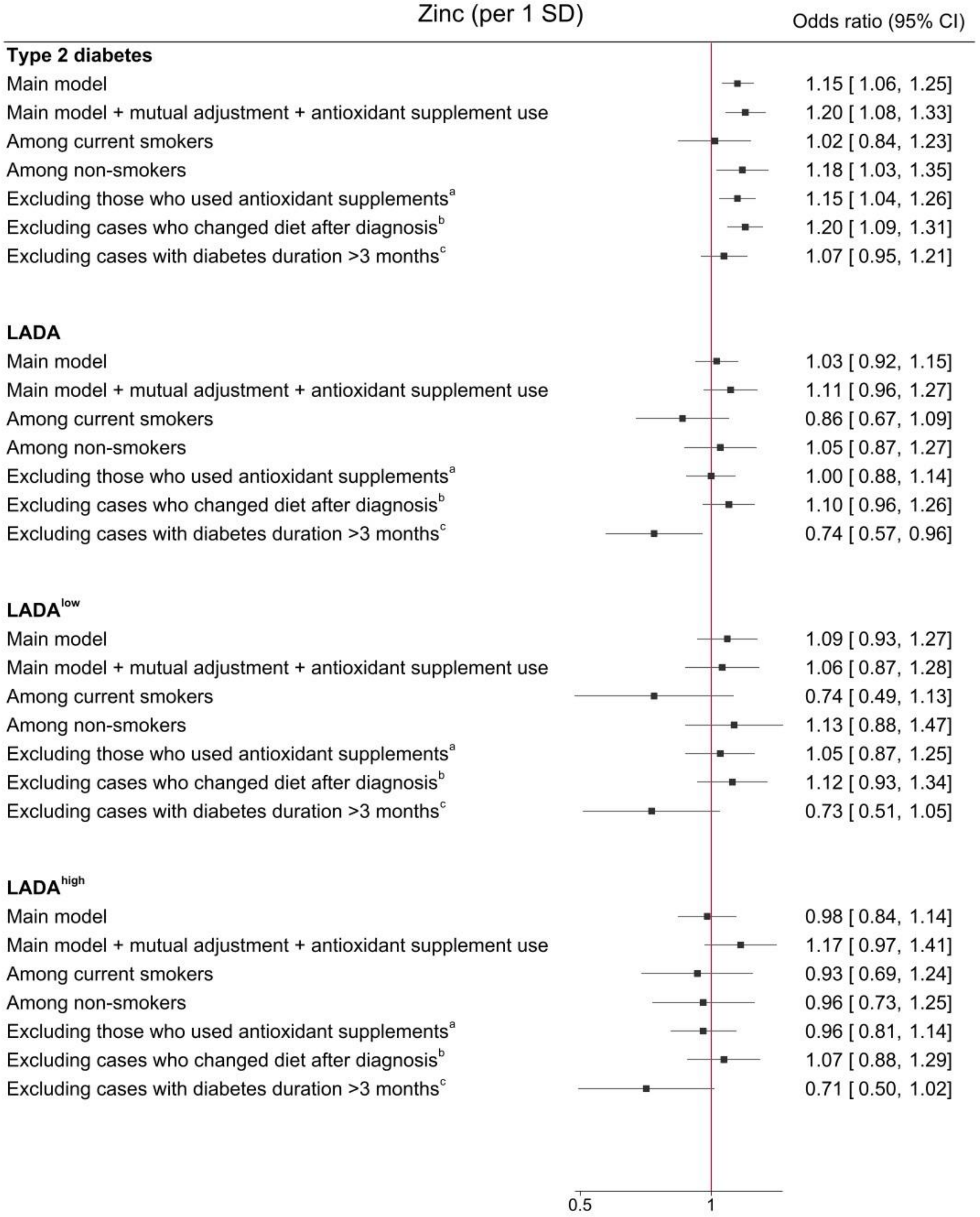

Figure S20. ORs and 95% CIs of type 2 diabetes, LADA, LADA^{low}, and LADA^{high} in relation to 1 SD higher zinc intakes, in different sensitivity analyses. Main model: adjusted for age, sex, education, physical activity, smoking, intake of alcohol, family history of diabetes, BMI, energy intake, tertiles of fatty fish (except for selenium), red meat (except for selenium and zinc), sweetened beverages, and coffee intake, and history of cardiovascular diseases or hypertension. [a] n=1,240 excluded individuals. [b] n=758 excluded individuals. [c] n=1,826 excluded individuals.
